# Microbial enrichment and gene functional categories revealed on the walls of a spent fuel pool of a nuclear power plant

**DOI:** 10.1371/journal.pone.0205228

**Published:** 2018-10-04

**Authors:** Rosane Silva, Darcy Muniz de Almeida, Bianca Catarina Azeredo Cabral, Victor Hugo Giordano Dias, Isadora Cristina de Toledo e Mello, Turán Péter Ürményi, August E. Woerner, Rodrigo Soares de Moura Neto, Bruce Budowle, Cristina Aparecida Gomes Nassar

**Affiliations:** 1 Instituto de Biofísica Carlos Chagas Filho, Universidade Federal do Rio de Janeiro, Rio de Janeiro, Brazil; 2 Escola Politécnica & Escola de Química, Universidade Federal do Rio de Janeiro, Rio de Janeiro, Brazil; 3 Center for Human Identification, University of North Texas Health Science Center, Fort Worth, United States of America; 4 Instituto de Biologia, Universidade Federal do Rio de Janeiro, Rio de Janeiro, Brazil; 5 Center of Excellence in Genomic Medicine Research (CEGMR), King Abdulaziz University, Jeddah, Saudi Arabia; Leibniz Institut - Deutsche Sammlung von Mikroorganismen und Zellkulturen GmbH, GERMANY

## Abstract

Microorganisms developing in the liner of the spent fuel pool (SFP) and the fuel transfer channel (FTC) of a Nuclear Power Plant (NPP) can form high radiation resistant biofilms and cause corrosion. Due to difficulties and limitations to obtain large samples from SFP and FTC, cotton swabs were used to collect the biofilm from the wall of these installations. Molecular characterization was performed using massively parallel sequencing to obtain a taxonomic and functional gene classification. Also, samples from the drainage system were evaluated because microorganisms may travel over the 12-meter column of the pool water of the Brazilian Nuclear Power Plant (Angra1), which has been functioning since 1985. Regardless of the treatment of the pool water, our data reveal the unexpected presence of Fungi (Basidiomycota and Ascomycota) as the main contaminators of the SFP and FTC. Ustilaginomycetes (Basidiomycota) was the major class contributor (70%) in the SFP and FTC reflecting the little diversity in these sites; nevertheless, Proteobacteria, Actinobacteria, Firmicutes (Bacilli) were present in small proportions. Mapping total reads against six fungal reference genomes indicate that there is, in fact, a high abundance of fungal sequences in samples collected from SFP and FTC. Analysis of the ribosomal internal transcribed spacer (ITS) 1 and 2 regions and the protein found in the mitochondria of eukaryotic cells, cytochrome b (cytb) grouped our sample fungi in the clade 7 as *Ustilago* and *Pseudozyma*. In contrast, in the drainage system, Alphaproteobacteria were present in high abundances (55%). The presence of *Sphingopyxis*, *Mesorhizobium*, *Erythrobacter*, *Sphingomonas*, *Novosphingobium*, *Sphingobium*, *Chelativorans*, *Oceanicaulis*, *Acidovorax*, and Cyanobacteria was observed. Based on genomic annotation data, the assessment of the biological function found a higher proportion of protein-coding sequences related to respiration and protein metabolism in SFP and FTC samples. The knowledge of this biological inventory present in the system may contribute to further studies of potential microorganisms that might be useful for bioremediation of nuclear waste.

## Introduction

There is consideration of extending the use of many Nuclear Power Plants (NPP) from 40 to 60 years of operation. The nuclear power plant Angra 1 in Rio de Janeiro, Brazil has a pressurized water reactor and after 12 months, part of the fuel (Uranium-235) in the core of the reactor is replaced. The spent fuel remains thermal active and radioactive and is transferred through a fuel transfer channel (FTC) to racks at the bottom of a spent fuel pool (SFP). The pool water contains Boron, a neutron-absorbing element to avoid critical heating of the pool. FTC and SFP are two of the locations to be studied for integrity and secure storage capacity of the fuel elements when considering extension of the duration of operation of the NPP. To ascertain security, operators search for any small leaks through micro-cracks in the liner and the drainage system by periodic inspection and through the SFP alarm level in the control room. Detecting microorganisms in the liner is important because their growth may cause deterioration of the NPP facility through microbial-induced corrosion (MIC) [1]. The water that eventually passes through the liner cracks is directed to the drainage system, and the volume is measured periodically to evaluate the waste. The pool water is oligotrophic and radioactive and does not provide a favorable environment to support life. However, there is a possibility of proliferation of algae, fungi, and bacteria, which together may form a biofilm [2]. Microorganisms that can grow under the harsh conditions of the NPP potentially can accumulate radionuclides and thus may be useful for bioremediation of radioactive environments [3–6]. Therefore, an in-depth analysis of the water quality also should include periodic assessment of possible growth of microorganisms in the pool and the inner coating of the drainer tubes. However, few studies have been conducted on microorganisms in NPP [3,7,8]. One study using analysis of 16S rRNA identified a diverse group of bacteria [8] and another isolated six bacterial taxa in the SFP pool water samples with radio-tolerance and biofilm-forming capabilities, but no species characterization was provided [3]. Also, an autotrophic freshwater green microalga was isolated by culture methods from a cooling pool of a nuclear reactor containing spent nuclear fuels in France [9]. This extremophile of the genus *Coccomyxa* is resistant to UV and gamma radiation. To estimate diversity and resolve taxa, improved tools to describe microbial diversity were described [10,11]. Also, the culture approach favors the microorganisms that were able to grow in a particular nutrient. Therefore, better resolving, unbiased methods are needed to characterize and describe biodiversity, especially in hazardous environments. Given the difficulties and limitations to collect samples from the SFP, small samples from the wall of this installation were collected. Subsequently, massively parallel sequencing (MPS) was applied for metagenomic sampling to analyze the microorganisms present in the walls of the SFP and FTC. Also, microorganisms in the drainage system may have traveled over the 12-meter column of the water pool where irradiated fuel elements with high activity (0.416 Gy/h) are stored. In addition to the inventory of microorganisms, biological function present in the system was assessed.

## Materials and methods

### Study location

The Almirante Álvaro Alberto Nuclear Power Plant (Angra 1) is located south of Rio de Janeiro city, in Rio de Janeiro State (23° 00′ 31.42″ S, 44° 27′ 26.87″ W), Brazil. SFP and FTC are diagramed in **S1 Fig**. Physicochemical parameters of the water, such as temperature, conductivity, pH, and concentration of boron, chloride, fluoride, silica, and sulfate were performed by routine analysis of the SFP and did not vary in the period of the sample collection.

### Sample collection

Samples were collected under Radiological Working License No. 543. Samples were collected with the aid of sterile cotton swabs with plastic rod tubes on 12/09/2015 between 09:45 h and 11:35 h. The stem of the swab was held at its lower half of the swab and rubbed firmly, from top to bottom and vice versa, then rotated approximately 180 degrees and the procedure was repeated. After collection, cotton swabs were inserted into the tube. Fourteen samples were collected in four different locations: Four samples were taken from the surface of the liner of the FTC and four others from the SFP, both at 15 cm below the water level. Six samples were collected from the drainage system, three from dried drainer (Dry Drainers–DD) and three from drainer tubes that were wet at the time of the collection (Wet Drainers–WD). Radionuclide measurements were performed using the 40 FH GL Series 22676 Radiometer equipment.

### DNA extraction

Collected cotton swabs (N = 3 or 4) were pooled to be representative of the four distinct locations. These locations were (1) the liner of the spent fuel pool (SFP); (2) the liner of the fuel transfer channel (FTC); and the drainage system identified as (3) dry (DD) and (4) wet drainers (WD). Nucleic acid extraction was performed for all cotton swabs. Briefly, 500 μl of TE buffer (TRIS-HCl 10 mM, EDTA 1 mM, pH 7.5 containing freshly added 0.2 g/mL proteinase K and 0.1% SDS) was added to the cotton swabs and incubated for 16 h at 37°C. An equal volume of the solution of phenol-chloroform equilibrated with TRIS buffer (10 mM, pH 8.0) was added to the mixture and vortexed for 30 seconds. DNA present in the aqueous phase was precipitated by the addition of 0.3 M (pH 5.5) sodium acetate and 2.5 volumes of ethanol followed by centrifugation at 13,000 rpm in a microfuge. The DNA was suspended in TE-4 solution (TRIS-HCl 10 mM, EDTA 0.1 mM, pH 7.5) quantified by spectrophotometry (NanoDrop 2000, ThermoScientific, USA) and stored at -20°C.

### Library construction and Next Generation Sequencing

The DNA libraries were prepared using the Ion Xpress Plus Fragment Library Kit as recommended by the manufacturer (Thermo Fisher, San Francisco, CA). The pooled samples from SFP, FTC, DD and WD were used to generate four libraries. A total of 100 ng of DNA from each sample was enzymatically fragmented for two minutes using Ion Shear kit according to the manufacturer's instructions (Life Technologies, Carlsbad, CA). Fragmentation patterns were evaluated by electrophoresis microfluidics using DNA 1000 Kit in a Bioanalyzer (Agilent Technologies, Santa Clara, CA) and quantified using the ds DNA HS assay Kit Qubit fluorometer (Invitrogen Co. Carlsbad, CA). Sequencing was performed using a 318 chip (Life Technologies, Carlsbad, CA) in the Ion Torrent PGM platform (Life Technologies, Carlsbad, CA).

### Data analysis

Sequence data were exported in FASTQ format by the Torrent Suite software and uploaded to the MG-RAST server (http://metagenomics.anl.gov/) on May 2016. Processing of shotgun reads was performed using the metagenomics pipeline [12] with default options for initial quality control (QC) filtering of raw reads. Low quality (Phred<15) and duplicated sequences were removed. The maximum e-value threshold of 1e-5, minimum alignment length of 50 bp and minimum of identity cutoff of 60% were used for functional annotation and classification. Venn diagrams were prepared at http://bioinfogp.cnb.csic.es/tools/venny/index.html. GC-pattern for each sequenced sample was evaluated through GC distribution among the reads to determine the heterogeneity of the DNA sequences on the online server MG-RAST. The search for fungal and bacterial genera was performed on the online server MG-RAST, using the M5NR database. This database is an integrated database that comprises sequences from NCBI, KEGG, EBI and other databases. Functional category identification was performed using reads matching to the M5NR database [13], which is based on genomic annotation data that are curated in a set of functional roles (SEED system) [14]. Functional category analysis of the genes found in the metagenomic samples was performed to investigate the metabolic capabilities of the microorganisms present in the SFP, FTC and drainage system. The taxonomic analysis was performed to determine the distribution of prokaryotic and eukaryotic organisms in the sampled areas. All the reads assigned to a taxon were counted, and their relative abundance was established. The potential of the metagenomic sequencing reads encompassing partial or entire genomes was tested by mapping these reads to reference genomes. Mapping was performed using CLC Genomics Workbench v8.5.1 with the following parameters: no global alignment; 0.8 similarity fraction; 2 of mismatch cost; 0.5 of length fraction. Assembling reads to a contig was performed using the parameters: reads were mapped back to contigs, using mismatch cost = 2; insertion cost = 3; deletion cost = 3; length fraction = 0.5; similarity fraction = 0.8. Subset of the variants calls, or ploidy variant detection was performed using the following parameters: fixed ploidy = 2; required variant probability = 90%; minimum coverage = 10; minimum count = 2: minimum frequency (%) = 20%. To characterize the fungal species, the barcode analysis was performed using ITS. The search for ITS consensus sequences was performed using Blastn against our sequence database of contigs using ITS sequences from different fungi as query sequences. An alignment of ITS sequences from 25 members of Ustilaginales selected from the NCBI database was performed using clustalW progressive alignment methods. The algorithm computes a rough distance matrix between each pair of sequences using pairwise sequence alignment scores. Progressively more distant groups of sequences are aligned until a global alignment is obtained. MEGA 6 was used to generate a phylogenetic tree by Maximum Likelihood, using Kimura 2-parameter, gamma distribution and evolutionarily invariable sites (K2+G+I) as nucleotide substitution model, and bootstrap of 500 replicates. The cytb phylogenetic tree was built using amino acid data, applying three different algorithms. Mega 6 was used to find the best-fit substitution model. For the cytb analysis, the best one, with smaller BIC (Bayesian Information Criterion) was mtREV24, described as General Reversible Mitochondrial [15], and it was used for building a Maximum Likelihood tree. The JTT best-fit model [16], was used for generating a Neighbor-Joining tree. No substitution model was used to generate a Maximum Parsimony tree.

## Results

### Physicochemical and radiochemical parameters of the spent fuel pool and transfer channel

Measurements of physical and chemical parameters of the spent fuel pool and transfer channel SFP are described in **Table 1**. Radiation dosage at the collection points in the SFP and FTC was 2μSv/h. The concentration of radionuclides for each cotton swabs used to collect samples was recorded previously in a conservative manner by spectrometry containing ^51^Cr, ^58^Co, ^60^Co, ^137^Cs radionuclides. Total activity was 5.122 x 10^2^Bq/g.

**Table 1:** Physical and chemical parameters of the water of the spent fuel pool and fuel transfer channel.

Parameters	
Specific conductivity	<20 μS / cm
pH	4.0–4.7
Boron	2,500 mg/L—3,000 mg/L
Chloride	<150 μg/L
Fluoride	<150 μg/L
Silica suspended solids	<50 μg/L
Sulfate	<150 μg/L
Temperature	35°C

### Metagenomic sequencing and taxonomic analysis of microorganisms present in the spent fuel pool, transfer channel, and drainage systems

A total of 2,741,680 reads, obtained from sequencing, was uploaded to the online service MG-RAST for metagenomic analysis. Taxonomic classification results showed that parts of the microbial communities of fungi (class) and bacteria (phylum) are shared among the NPP sites, SFP, FTC, DD and WD (**S2 Fig**), but the relative abundance of microbial taxa distribution is different (**Table 2).**

**Table 2:** Relative abundance of microbial taxa among four sample types: Spent fuel pool (SFP), Fuel transfer channel (FTC), Dry drainer (DD), and Wet drainer (WD). Values correspond to percentage. In bold, the highest value for each group.

Microbial distribution (%)					
		SFP	FTC	DD	WD
**Domain**	Archaea	*	*	0.3	0.2
	Bacteria	3.6	3.6	**85.2**	**84.6**
	Eukaryota	**94.7**	**94.3**	0.7	0.5
	Viruses	**	*	*	0.5
	Others/unassigned/unclassified sequences	1.8	2.1	13.9	14.2
		100	100	100	100
**Phylum**	Proteobacteria	2.4	2.3	**76.4**	**81.1**
	Actinobacteria	0.7	0.6	8.1	2.5
	Firmicutes	0.5	0.6	3.9	1.9
	Bacteroidetes	0.4	0.5	3	8.4
	Acidobacteria	*	*	1.5	0.8
	Cyanobacteria	0.1	0.1	1.1	0.6
	Chloroflexi	*	*	1	0.5
	Planctomycetes	*	*	0.9	0.3
	Deinococcus-Thermus	*	*	0.4	0.2
	Verrucomicrobia	*	*	0.4	0.2
	Chlorobi	*	*	0.3	0.3
	Chlamydiae	**	**	0.2	0.4
	Euryarchaeota	*	*	0.3	0.2
	Ascomycota	10.9	10.8	0.4	0.2
	Basidiomycota	**79.2**	**79.3**	*	*
	Others/unassigned/unclassified sequences	5.8	5.8	2.1	2.4
	Total	100	100	100	100
**Class**	Actinobacteria	0.7	0.6	8.1	2.5
	Alphaproteobacteria	0.7	0.7	**54.5**	**57.6**
	Bacilli	0.4	0.4	2.4	0.9
	Betaproteobacteria	0.7	0.6	8.8	15.2
	Clostridia	0.1	0.1	1.4	0.9
	Deltaproteobacteria	0.1	0.1	2.4	1.5
	Flavobacteriia	0.3	0.2	1.5	2.9
	Gammaproteobacteria	0.8	0.7	10.6	6.6
	Sphingobacteriia	*	*	0.5	3.2
	Agaricomycetes	2.4	2.3	*	**
	Eurotiomycetes	1.8	1.8	*	*
	Exobasidiomycetes	2.3	2.5	**	**
	Saccharomycetes	1.9	1.7	*	*
	Schizosaccharomycetes	1.9	1.8	**	**
	Sordariomycetes	4.6	4.7	0.2	0.1
	Tremellomycetes	3.3	3.9	***	***
	Ustilaginomycetes	**70.9**	**70.2**	*	**
	Others/unassigned/unclassified sequences	7.1	7.6	9.6	8.6
	Total	100	100	100	100
**Genus**	*Sphingopyxis*	***	***	2.1	**7.5**
	*Mesorhizobium*	***	***	**7.1**	**6.9**
	*Erythrobacter*	***	***	**4.2**	3.9
	*Sphingomonas*	***	***	3.0	3.7
	*Novosphingobium*	***	***	3.6	3.4
	*Sphingobium*	***	***	2.4	2.4
	*Chelativorans*	***	***	2.6	2.1
	*Pseudomonas*	***	***	3.8	1.0
	*Oceanicaulis*	***	***	2.0	*
	*Acidovorax*	0.2	0.2	*	2.3
	*Ustilago*	**71.9**	**72.2**	***	***
	*Cryptococcus*	3.9	3.3	***	***
	*Schizosaccharomyces*	1.7	1.8	***	***
	*Malassezia*	1.4	1.6	***	***
	*Gibberella*	1.3	1.4	***	***
	*Neurospora*	1.1	1.2	***	***
	*Tilletia*	1.1	0.8	***	***
	Others	17.3	17.7	69.2	66.9
	Total	100	100	100	100

* less than 0.09%

**less than 0.009%

***less than 0.0009%.

Unassigned/unclassified sequences were computed together with other minor taxa.

Bacteria comprised 85% of the microorganisms in these samples and Basidiomycota and Euryarchaeota in the drainage system were minor contributors (less than 0.7%). Proteobacteria represented 76.4% (DD) and 81.1% (WD). This group was followed by Actinobacteria (8.1% and 2.5%), Bacteroidetes (3.0% and 8.4%), and Firmicutes (3.9% and 1.9%), in DD and WD samples, respectively. Regarding the class of bacteria, Alphaproteobacteria (54.5% and 57.6%) was the most abundant in DD and WD sampled locations, respectively. The major genera of Alphaproteobacteria were *Sphingopyxis* (2.1% and 7.5%), *Mesorhizobium* (7.1% and 6.9%), *Erythrobacter* (4.2% and 3.9%), *Sphingomonas* (3.0% and 3.7%), *Novosphingobium* (3.6% and 3.4%), *Sphingobium* (2.4% and 2.4%), *Chelativorans* (2.6% and 2.1%), and *Oceanicaulis* (2.0% and < 0.1%), in DD and WD samples respectively. *Pseudomonas*, a genus of Gammaproteobacteria, *Acidovorax* a genus of Betaproteobacteria and fungal constituents, such as *Sordaria*, *Ustilago*, and *Aspergillus*, were found in minor proportions in DD and WD samples. Conversely, in SFP and FTC samples, the major contributors were eukaryotes (around 94%), in which the fungi Basidiomycota (79%), and Ascomycota (11%) were the major phyla. The dominant class was Ustilaginomycetes making up 70.9% and 70.2% of the SFP and FTC samples, respectively, followed by Sordariomycetes (4.6% and 4.7%), Tremellomycetes (3.3% and 3.9%), Agaricomycetes (2.4% and 2.3%), Exobasidiomycetes (2.3% and 2.5%), Saccharomycetes (1.9% and 1.7%) and Schizosaccharomycetes (1.9% and 1.7%). Bacteria constituted only 3.6% of the phyla in the SFP and FTC samples. *Ustilago* represented 72% of the fungal genera observed in the SFP and FTC. Other genera of fungi were found at lower percentages (less than 4%) in both samples. The detected fungi included *Cryptococcus* (Tremellomycetes), *Schizosaccharomyces* (Schizosaccharomycetes), *Malassezia* (Malasseziomycetes), *Gibberella* (Sordariomycetes), *Neurospora* (Ascomycetes) and *Tilletia* (Exobasidiomycetes). Bacteria constituted less than 3.5% of the taxa in SFP and FTC, and the genus *Acidovorax* was present in a very low percentage. Further analysis focused on the fungi as they were far more abundant than the bacteria in the FTC and the SFP. The presence of the dominant *Ustilago* (Ustilaginomycetes) in the SFP and FTC (**Table 2**) was evaluated by mapping the total reads against available fungal genome sequences. *Ustilago* and closely related fungi genomes were compared to the total reads obtained from SFP and FTC. Total reads from SFP (80,270,543 bp) and FTC (78,047,151 bp) were mapped against the available Ustilaginales fungal sequences (**Table 3**).

**Table 3:** Mapping of the metagenomic reads to closely related Ustilaginales fungi sequences.

References (BioProjects)	Myosarcoma maydis* (PRJNA1446) GC: 53.9%		*Pseudozyma hubeiensis SY62* (PRJDB993) GC: 56.5%		*Moesziomyces aphidis* (PRJNA215967) GC: 60.9%		*Kalmanozyma brasiliensis* (PRJNA292598) GC: 58.1%		*Ustilago sculenta (*PRJNA263330) GC: 54.4%		*Sporisorium reilianum SRZ2* (PRJNA64587) GC:59.5%			
Genome size	19,643,891 bp		18,435,583 bp		17,921,702 bp		17,325,407 bp		20,196,985 bp		18,334,746 bp			
**Sample**	SFP	FTC	SFP	FTC	SFP	FTC	SFP	FTC	SFP	FTC	SFP	FTC	DD	WD
**Mapped reads %**	16.12	16.49	19.72	19.77	20.71	21.41	20.08	20.04	20.51	20.61	22.24	22.99	0.81	0.38
**Not mapped reads %**	83.88	83.51	80.28	80.23	79.29	78.59	79.92	79.96	79.49	79.39	77.76	77.01	99.19	99.62

Total reads (bp): **SFP**: 80,270,543; **FTC**: 78,047,151; **DD**: 128,740,448; **WD**: 140,814,771.

*, species previously known as *Ustilago maydis*.

**Fig 1:**
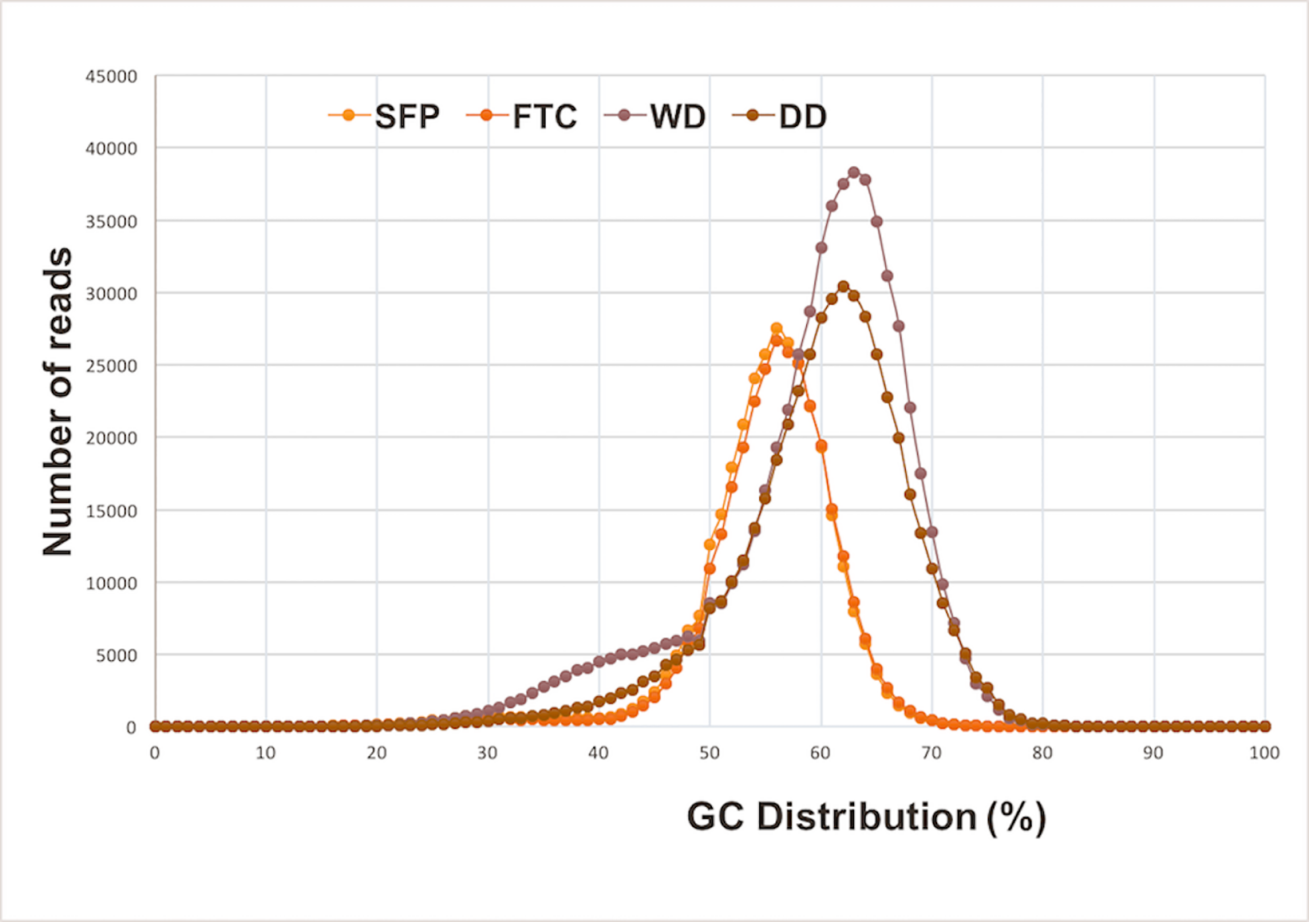
GC-content distribution of the metagenomes reads sampled from NPP sites. GC-content (%) for the total number of reads sequenced in SFP, FTC, DD and WD.

The highest percentage of mapped reads (22% and 23%) from the SFP and FTC were obtained with *Sporisorium reilianum* when compared with the others fungal genomes *Mycosarcoma maydis* (previously known as *Ustilago maydis*) [17–19], *Pseudozyma hubeiensis*, *Moesziomyces aphidis*, *Kalmozyma braziliensis*, *U*. *esculenta*. On the other hand, only 0.81% and 0.38% of total reads from samples DD and WD mapped to the *S*. *reilianum* genome. Because environmental metagenome types usually have a distinct GC-pattern, we analyzed the GC-content of our metagenomics reads (**Fig 1**). A similar GC distribution was observed for SFP and FTC sequencing reads peaking at 52 to 60%. In contrast, DD and WD samples peaked at 60 to 70% of GC-content.

To further investigate the diversity of fungal and bacterial elements in our samples, the assembled reads into contigs from SFP, FTC, DD and WD were compared separately. **Table 4** shows the contigs assembly data from SFP and FTC reads. They were, on average, bigger (772 bp and 647 bp, respectively) and smaller in number (19,986 and 25,688), when compared to DD and WD samples reads (482 bp and 436 bp; 43,391 and 46,188 contigs).

**Table 4:** Contig assembly statistics.

Feature	SFP	FTC	DD	WD
N75 (bp)	579	501	346	313
N50 (bp)	930	854	540	483
N25 (bp)	1,504	1,417	1,083	887
Minimum (bp)	165	100	81	18
Maximum (bp)	14,560	11,241	18,195	20,580
Average (bp)	772	647	482	436
Contig Count	19,986	25,688	43,391	46,188
Total (bp)	15,428,667	16,631,355	20,895,983	20,137,239

**Fig 2:**
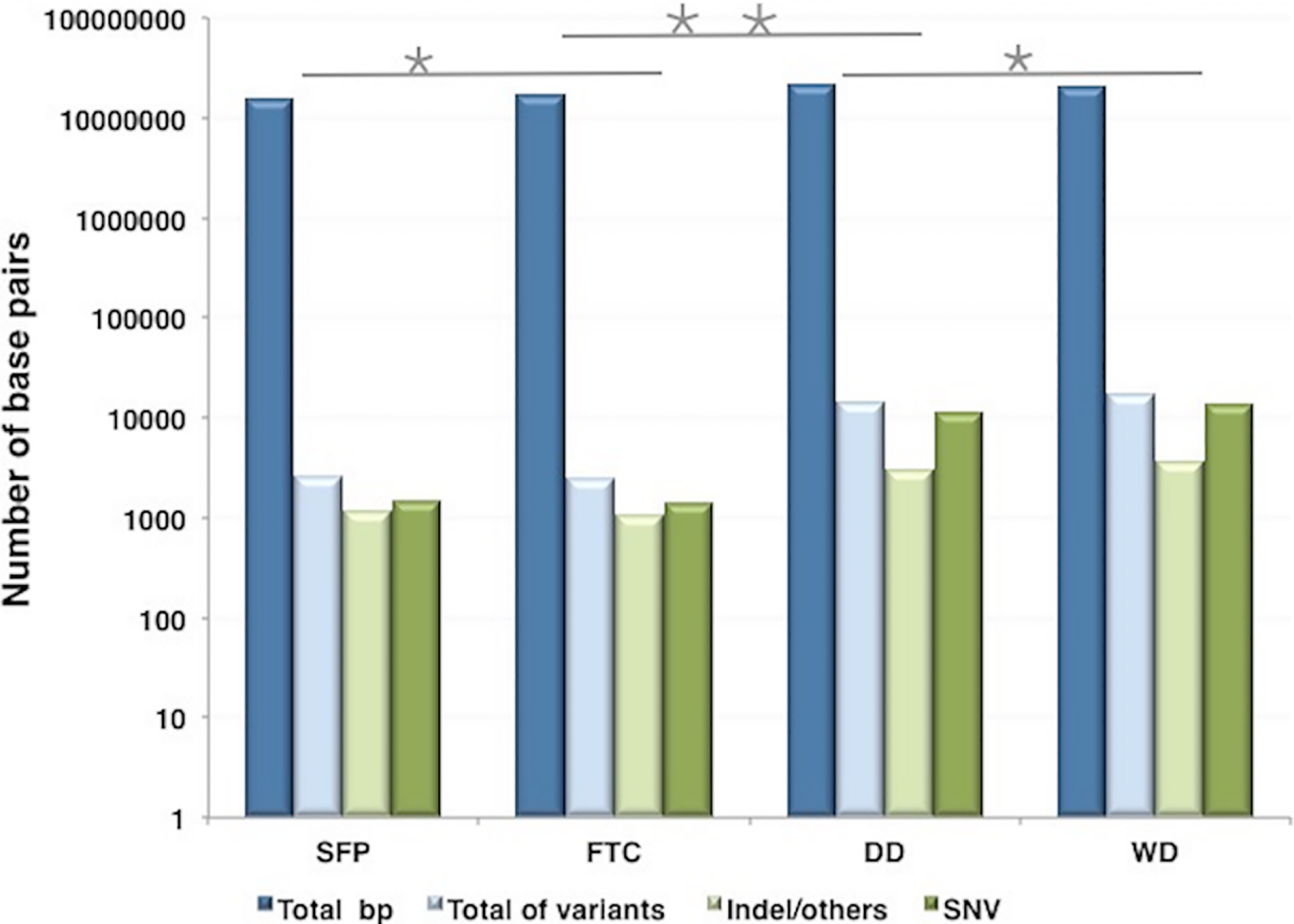
Occurrence of nucleotide sequence variation among contigs assembled from SFP, FTC, DD and WD sequence reads. Total base pairs are represented as dark blue bars; Total nucleotide variation as light blue bars; indel, and others as a light green bar; and single nucleotide variation as dark green. * Two-tailed p-value = 0.690; ** p<0.0001; Chi-square with Yates’ correction.

Lower sequence diversity was observed in SFP and FTC. In fact, 23% of the sequences organized in contigs from SFP were mapped to the 23 chromosomes of *S*. *reilianum* meaning i.e., 3,531,104 bp, from a total of 15,428,667 bp (data not shown). To investigate the diversity of the microorganisms present in the samples the nucleotide variation within all the assembled contigs was analyzed. **S2 Table** shows the consensus length, the total read counts and the average coverage of each of the 19,986; 25,688; 43,391 and 46,188 contigs assembled from reads obtained from SFP, FTC, DD, and WD, respectively. A subset of the variant calls was performed to verify the diversity of the consensus sequences for each contig considering a coverage of at least 10X for each nucleotide position with more than 10% of frequency **(Fig 2)**.

**Fig 3:**
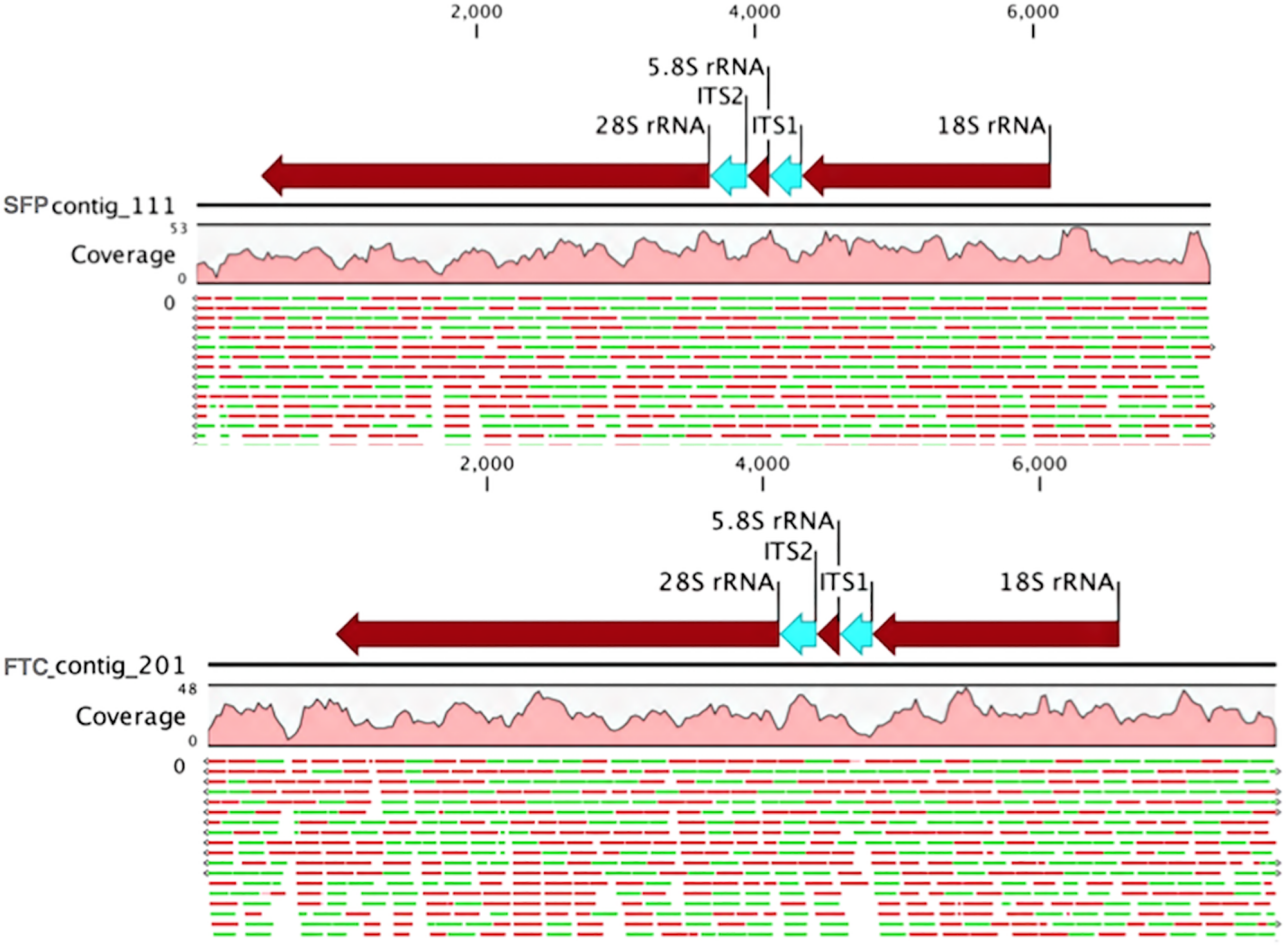
Diagram of ribosomal containing-contigs. Reads from each sampled area (SFP and FTC) assembled in a consensus sequence encompassing the ribosomal gene cluster (brown arrows) and ITS1 and ITS2 (blue arrows). Contigs 111 and 201 represent the assembled forward and reverse reads (green and red lines at the bottom of each contig); Solid pink hills account for the nucleotide coverage along the contigs.

We found 2,597 variants in SFP contigs, of which 1,455 were single nucleotide variation (SNV) and 1,142 other variants including deletions and insertions) and 2,420 variants in FTC contigs (1,388 SNV, and 1,037 variants including, deletions and insertions). In contrast DD and WD contigs had 13,726 variants (10,827 SNV) and 16,917 variations (13,437 SNV), respectively. The number of the contigs in samples DD and WD were roughly twice as many as in the SFP and FTC, but the variants found in these contigs were 4 to 5 times more abundant in DD and WD samples. The diversity of microorganisms found in DD and WD was higher compared to SFP and FTC samples. To characterize the fungal species, present in SFP and FTC, the internal transcribed spacer (ITS) was analyzed in our metagenomics reads. One contig for each sampled area (SFP and FTC) was obtained that encompasses the ribosomal gene cluster. In the SFP contig archive, contig 111 was found which has a consensus length of 7,268 bp, 18,203 read counts and 28X of average coverage. In the FTC contig archive, contig 201 was found which has a consensus length of 7,720 bp, covered by 1,148 reads and 25X of average coverage (**Fig 3**).

**Fig 4:**
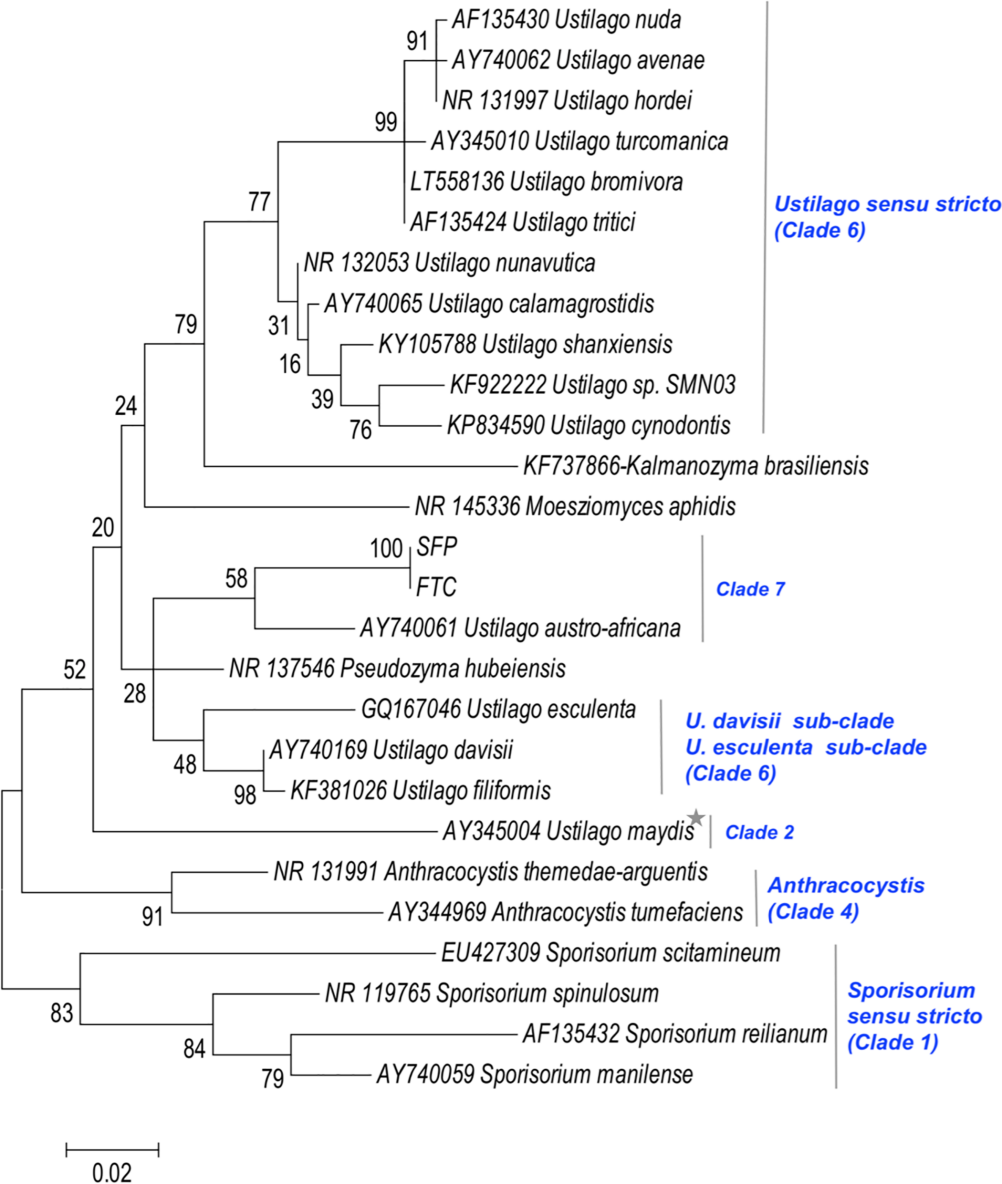
Phylogenetic tree of Ustilaginales members obtained after pair-wise DNA sequence alignment of 630 bp encompassing ITS1 and 2 and 5.8S rRNA using a maximum likelihood phylogenetic approach. Bootstrap values for each node are indicated. GenBank IDs are displayed with the species name. Clades are indicated according Wang *et al*. 2015. * *Mycosarcoma maydis*.

These two contigs containing the genes for 18S, 5.8S and 28S ribosomal rRNA and the respective ITS1 and ITS2 were aligned to closely related Ustilaginomycetes fungi (**S3 Table**). No nucleotide variations were observed in the aligned region, containing ITS1 and ITS2 and 5.8S rRNA, of the two contigs (201 and 111). Alignment of an ML phylogenetic tree was based on a 630-bp region of the ITS1-5.8S rRNA-ITS2 sequences of 25 members of the order Ustilaginales selected from the NCBI ITS region from fungi strains and reference material. The ML phylogenetic tree (**Fig 4**) shows the relationship of the fungus in our sample. The contig consensus sequences of SFP and FTC were positioned among the clade of *Ustilago austro-africana* (Clade 7). The ML tree of our SFP and FTC samples using ITS barcoding were grouped in the clade of *Ustilago austro-africana* (Clade 7).

Also, the phylogenetic tree of cytochrome oxidase protein (cytb), with available amino acid sequences from related fungi, confirmed grouping into the clade 7 with *Pseudozyma thailandica*, in all three topology algorithms (**S3 Fig**).

### Functional analysis of the microorganisms

**Fig 5:**
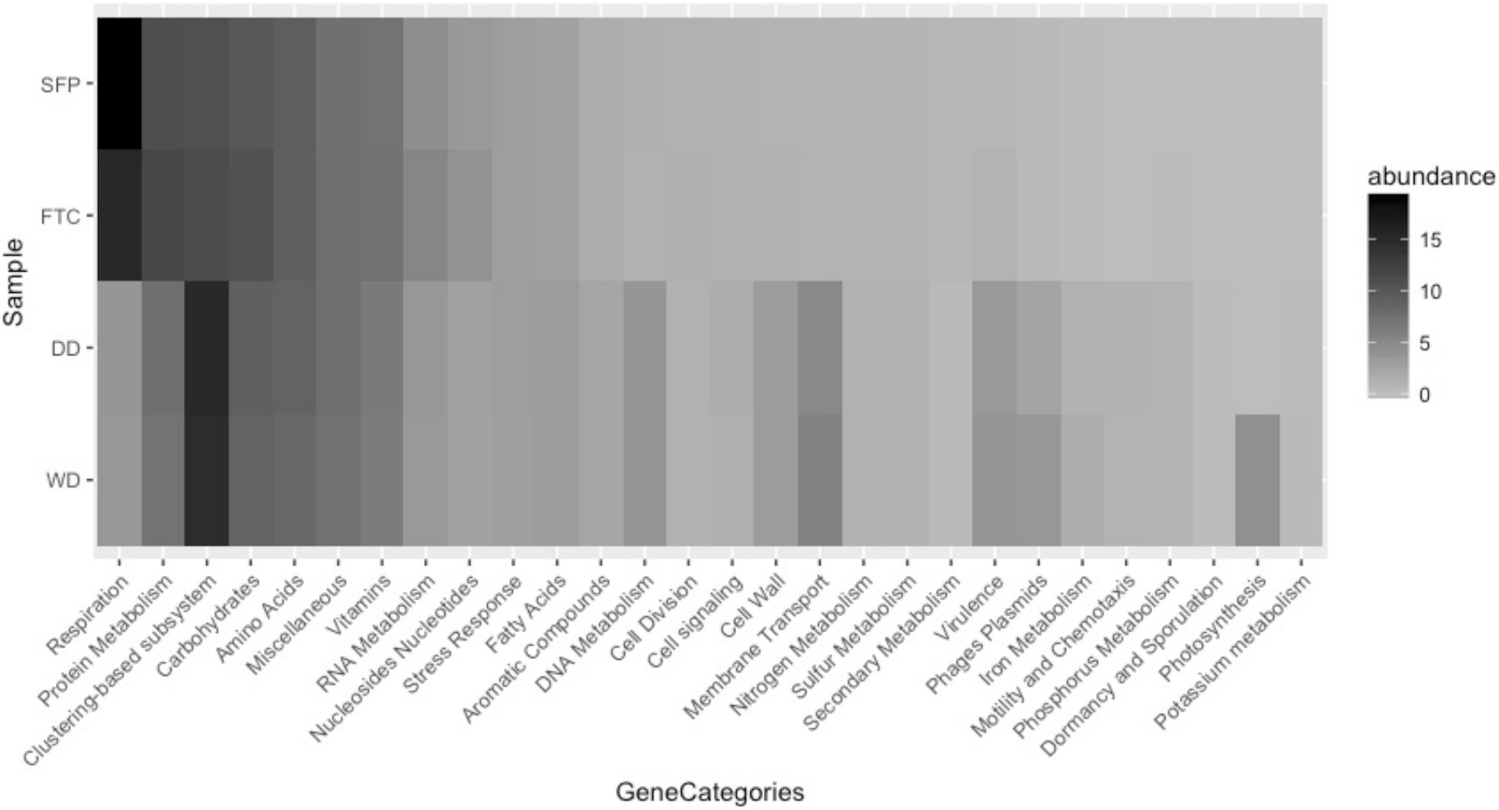
Relative abundance of the group of protein coding genes associated with functional categories. Each row denotes one area of study: spent fuel pool (SFP), Fuel transfer channel (FTC) drainage system, dry drainer tubes (DD) and wet drainer tubes (WD). Online server MG-RAST generated the set of functional gene groups. Clustering-based subsystems represent genes that indicate that they belong together, but no function is associated with them yet.

The metabolic capabilities of the microorganisms present in the SFP, FTC and drainage system are shown in **Fig 5.** Relative abundance of functional gene categories from SFP, FTC, DD and WD indicate that protein coding sequences involved in the amino acids and derivatives pathway, carbohydrates, respiration, RNA metabolism and metabolism of aromatic compounds, were present.

Relative abundance indicate that the functions related to DNA metabolism, cell wall, membrane transport, virulence, and defense were more abundant in the WD and DD samples than in the FTC and SFP samples. In contrast, a higher number of protein sequences related to respiration and protein metabolism (**Fig 5**) were associated with SFP and FTC samples. Detailed analysis of eukaryote genes such as cytochrome c oxidase, NADH dehydrogenase, succinate dehydrogenase cytochrome b560 subunit, mitochondrial precursor, ATP synthase chains are displayed on **S4 Table.** These genes are present as major components of the eukaryote oxidative phosphorylation (**S4 Fig**).

## Discussion

### Microbial diversity in spent fuel pool and fuel transfer channel in an acidic environment with boron

The data indicate that microorganisms from the wall of the SFP and FTC are part of a sophisticated, surface-attached community. The microorganisms found in these samples were collected in pH 4.0 to 4.7 at 35°C. The group of bacteria and archaea described here can be found in soil at pH 3.7 to 4.5 [20,21]. Indeed, some microbial populations from acidic forest soils are more tolerant to acidic cultivation conditions [22]. Fungi are capable of adapting to environments with diverse pH values [23] and transcriptional response induced by changes in the pH of the medium from neutral to acidic or to alkaline was reported in *Mycosarcoma maydis* [24]. Conserved signal transduction pathways mediate ambient pH sensing and adaptation in yeast and fungi for alkaline pH [25], but the mechanisms helping to cope with acidic pH are still unknown. Fungi produce organic acids, and therefore are capable of contributing to MIC. Ustilaginomycetes, which were found in high content in the SFP and FTC samples, under certain environmental conditions may be a source of organic acids [26] to the aerobic and anaerobic bacterial types, modifying the environment creating a favorable situation for the corrosion of the stainless steel [27]. Although, the bio-corrosion in these NPP installation has not been an actual problem due to the constant monitoring of the drainage system, the presence of these microorganisms may pose a threat to the integrity of the stainless-steel liner. Sulphide or sulphate-reducing bacteria (SRB) were not observed in the stainless-steel biofilm. These microorganisms can alter the local physical/chemical conditions and lead MIC and may modify the environment with the presence of metabolites on the stainless steel creating fissures [27,28].The observation that Proteobacteria (Alpha, Beta, Delta, and Gamma), Actinobacteria, Firmicutes (Bacilli), were present only in low proportions in the SFP and FTC may be due to the use of boron in the spent pool to capture neutrons. Boron is an essential element for human and animals [29] in which in a dose-dependent manner, between 0.77 and 1.545 mg/ml, has been known to have antibacterial effects [30]. The unexpected detection of Fungi (Basidiomycota and Ascomycota) as the major microbial contributor to the spent fuel pool and transfer channel suggests that they can grow in the presence of boron concentrations of 2,500 mg/L to 3,000 mg/L to have antibacterial effects explaining the low bacterial percentage numbers in the examined samples.

### Microbial diversity in spent fuel pool, fuel transfer channel, dry drainer, wet drainer samples and exposure to radiation

Our results indicated *Ustilago* (Ustilaginomycetes) as the dominant genus (around 72%) in the SFP and FTC. The GC distribution observed for SFP and FTC sequencing reads peaked at 52 to 60% which are consistent with the high fungal content in the SFP and FTC and were similar to the fungal reference genomes. Mapping the metagenomic reads to the available genome sequences among the Ustilaginales was insufficient to identify the genus of the fungus in our sample. Members of this order are facultative biotrophic basidiomycetes and occur in a variety of environments, and an effort was made to obtain consistency in the fungal systematics of Ustilagomycetes [31–37]. ITS barcoding analysis grouping the SFP and FTC fungus in the clade 7 of *Ustilago austro-africana* suggests that this fungus in our SFP and FTC samples belongs to the Ustilaginales according to Wang *et al*. 2015 [36]. In their tree, using LSU rRNA D1/D2 domains and ITS1 and 2 regions including 5.8S rRNA gene, the group described that clade 7 is composed of *Sporisorium veracruzianum*, *Pseudozyma thailandica*, *Macalpinomyces viridans*, *M*. *spermophora*, *Pseudozyma tsukubaensis* and *Ustilago austro-africana*. The cytb tree grouped our sample in the same clade as *Pseudozyma thailandica* confirming the correct group of fungi (clade 7). Additional research may include incorporating morphological characters, parasite population data, physiology, radio resistance characteristics to clarify the species position in the *Ustilago-Sporisorium* clade 7. These closely related fungi might share the same properties that are seen in *Ustilago* as being capable of growing in a radionuclide environment up to 6000 Gy [38,39]. Black molds growing in and around the Chernobyl Nuclear Power Plant have been reported to be radiotrophic [40].

Exposure to the ionizing radiation of Chernobyl may change the electronic properties of melanin and might enhance the hyphae growth towards sources of radiation [41]. Examination of fungi and bacteria at the same time allows the direct comparison of the abundance patterns of the different domains. Few numbers of members of Proteobacteria, Actinobacteria, Firmicutes, Bacteroidetes and Cyanobacteria were present in the SFP and FTC when compared to fungal abundance. These findings suggest that the microorganisms found in SFP and FTC, which are exposed to a uranium radiation and boron, may favor the growth of fungi. Analogous findings [42] associated the outgrowth of fungi over bacteria when uranium up to 1,500 ppm was added to culture media. *Paenibacillus* species were prevalent on LB plates containing 3 ppm of uranium but colony count decreased as concentration rose to 600 ppm. Some *Bacillus* and *Paenibacillus* were also isolated from high background radiation (2.9 μGy/h) from the Chernobyl nuclear power plant disaster area [43], suggesting that Firmicutes/Bacilli have abundant genera diversity, broad physiological characteristics and adaptation capabilities [44–46]. Similarly, regarding the drainage system, *Sphingopyxis*, *Mesorhizobium*, *Erythrobacter*, *Sphingomonas*, *Novosphingobium*, *Sphingobium*, *Chelativorans*. *Oceanicaulis*, Acidovorax, and *Cyanobacteria* were found in our metagenomic study. One can assume that the microorganisms identified in this area (DD and WD) have traveled over the 12-meter column of the water pool with 894 irradiated fuel elements stored with high activity 0.416 Gy/h are stored. It was reported that Actinobacteria, Flavobacteria, Firmicutes (*Bacillus*) and Proteobacteria as well as Deinococcus-Thermus and Cyanobacteria were identified in the areas after the Chernobyl accident, resisting an exposure of 4000 Gy/h [43,47]. One Microalgae isolated from a biofilm growing on a projector immersed 3 m below the surface water from the water pool used to store spent fuel elements in a research nuclear reactor in France was able to survive intense gamma-rays irradiation, up to 2,000 times the dose lethal to human [9].

Studies of the NPP of Cofrentes in Valencia, Spain, [7] identified microorganisms attached to the spent nuclear fuel pool wall using culture, PCR-DGGE, sequencing of 16S rRNA and LSU rRNA fungal identification system. Groups such as Betaproteobacteria, Actinomycetales, *Bacillus*, *Staphylococcus* and Trichocomaceae [7] were identified. Further studies in the same NPP [8,48,49], using standard culture methods, DGGE, and sequencing of 16S rRNA fragments found *Ralstonia*, *Pseudomonas*, *Burkholderia*, *Stenotrophomonas*, *Sphingomonas*, *Methylobacterium*, *Afipia*, *Bradyrhizobium*, *Rhizobium*, *Streptococcus*, *Staphylococcus*, *Mycobacterium*, *Nocardia*, and *Chryseobacterium* [40]. Subsequently, they were able to isolate *Bacillus* and *Stenotrophomonas* from the biofilm that was produced in 34 months by immersing stainless steel plates into the spent fuel pool, but on titanium plates, *Ralstonia* and *Mycobacterium* were the most present [8,49].

### Functional capacity of microorganisms in spent fuel pool and fuel transfer channel

Function analysis presented in this work provided information about the microbial metabolism in the collected samples. The distribution of gene functional categories was similar between SFP and FTC and between DD and WD, suggesting similar microbial populations in these areas. Genes related to carbohydrate, amino acid, and vitamin metabolisms were similarly abundant in all sampled areas and probably reflect housekeeping functions. In contrast, genes related to respiration and protein metabolism were more abundant in SFP and FTC, and genes related to DNA metabolism, cell wall, membrane, and transport were more abundant in DD and WD. Interestingly, genes related to photosynthesis were almost exclusively found in WD, and those related to motility, chemotaxis, iron, and phosphorus metabolisms were mostly observed in DD and WD. These differences may reflect varying microbial populations, which in turn may be a result of divergent metabolic requirements. In addition, the detection of eukaryotic gene sequences related to respiration (~15%) suggests an abundance of fungi in SFP and FTC. Eukaryote cytochrome c oxidase, NADH dehydrogenase, succinate dehydrogenase cytochrome b560 subunit, mitochondrial precursor, ATP synthase chains are the major components of the eukaryote oxidative phosphorylation and indicate that the functional gene category respiration (**S4 Fig)** which are present in the SFP and FTC are likely due to the high abundance of the fungi.

## Conclusion

This work contains relevant information about the microbial content of the water of the SFP and the monitoring drainage systems of a NPP. Metagenomic analyses using massively parallel sequencing can identify potential taxa at least to the genus level. This capability is a vast improvement over using a single biomarker such as 16S rRNA and allows better identification of microorganisms (both bacterial and archaea). Since the microorganisms were able to survive in a radioactive environment, they may be able to accumulate and concentrate radionuclides from water. Future work should focus on isolating these organisms, characterizing them better and determining their potential decontamination capabilities. The surprisingly high amount of sequences of fungal origin indicated that Ustilaginomycetes identified in the spent fuel pool and transfer channel suggests that these fungi are able to grow in a radioactive environment. Radiation levels were low in the samples collected from the pool wall, and no problems related to bio-corrosion materials were observed. Further genome and transcriptome analyses of the fungus may bring some understanding of what contributes to its persistence in the SFP and FTC. Our results were obtained without culture methods or PCR amplification of target genes. The use of cotton swabs was adequate for sampling material and the benefit of showing negligible levels of radionuclides (5.122 x 10^2^Bq/g). Therefore, collected samples could be combined and associated with success via a fast detection method to identify bacterial and fungal distribution in the SFP and FTC. This work also described functional genes related to respiration in SFP, contributing to understanding the NPP environment. Our results may be of value for further studies at other sites and to identify potential microorganisms that can be used for bioremediation of nuclear waste.

## Supporting information

S1 Table General information of reads (after quality and trimming processes) uploaded to MG-RAST server. (DOCX)

S2 Table Contig assembly coverage characteristics: Consensus length, the total read counts and the average coverage of each of contig from SFP, FTC, DD and WD. (XLSX)

S3 Table Alignment of ITS1 and ITS2 regions of close related Ustilaginomycetes Fungi: CLUSTAL multiple sequence alignment. (DOCX)

S4 Table Gene function associated with respiration found in the SFP sample. (DOCX)

S1 Fig Illustration of the Spent Fuel Pool (SFP) and Fuel Transfer Channel (FTC) at the Nuclear Power Plant (NPP) installation of Angra 1, Rio de Janeiro, Brazil. (a) SFP photograph, showing the spent nuclear fuel at the bottom of the pool; (b) scheme of the compound that stores the spent nuclear fuel, left the SFP; and to the right the FTC; (C) FTC photograph showing the robot arm, at the bottom, used to transfer nuclear fuel to the reactor or from the reactor to the SFP. SFP and FTC facilities are massive concrete structures with a corrosive resistant stainless-steel ASTM A240, type 304L liner material to guarantee structural quality. The nuclear power plant Angra 1 in Rio de Janeiro, Brazil has a pressurized water reactor (PWR) that uses 121 fuel elements (256 rods, 4 meters high each) of enriched Uranium-235. After 12 months, part of the fuel in the core of the reactor is replaced. The spent fuel remains thermal active and radioactive and is transferred through a fuel transfer tube to 1252 storage cells of super compact racks at the bottom of a spent fuel pool (SFP). The quality of the water must meet strict requirements of purity and clarity allowing operators to handle irradiated fuel elements.
(TIFF)

S2 Fig Microbial composition of bacteria and fungi shared among the four NPP sites. (TIFF)

S3 Fig cytb phylogenetic tree of 17 Ustilaginales members using three different algorithms: **A)** Neighbor Joining, **B)** Maximum Likelihood, and **C)** Maximum Parsimony, the General Reverse Mitochondrial as substitution model. Pair-wise sequence alignment of 94 amino acids of cytb.
(TIFF)

S4 Fig Kegg pathway: Energy metabolism: 00190 Oxidative phosphorylation. (TIFF)
