# Supplementary material for: Microbial enrichment and gene functional categories revealed on the walls of a spent fuel pool of a nuclear power plant
Source: PLoS One. 2018 Oct 4;13(10):e0205228. doi: 10.1371/journal.pone.0205228 (PMC6171911; doi:10.1371/journal.pone.0205228)
Supplement: S1 Table — (DOCX) [file pone.0205228.s001.docx]

**S1 Table:** General Information of reads (after quality and trimming processes) uploaded to MG-RAST server.

|  |  | **SFP** | **FTC** | **DD** | **DW** |
| --- | --- | --- | --- | --- | --- |
| Reads upload  MG-Rast | **Counts** | 78,766,670 bp | 79,454,713 bp | 142,259,007 bp | 164,522,032 bp |
|  | **reads** | 470,054 | 449,805 | 911,570 | 910,251 |
|  | **length** | 173 ± 33 bp | 176 ± 37 bp | 156 ± 78 bp | 180 ± 46 bp |
|  |  |  |  |  |  |
| Reads after processing  MG-Rast | **Counts** | 53,598,695 bp | 51,943,405 bp | 81,916,705 bp | 97,798,240 bp |
|  | **reads** | 327,158 | 313,639 | 486,413 | 605,771 |
|  | **length** | 163 ± 38 bp | 165 ± 41 bp | 168 ± 50 bp | 161 ± 50 bp |
|  | **% GC** | 55 ± 6 % | 55 ± 6 % | 60 ± 7 % | 59 ± 9 % |
|  |  |  |  |  |  |
| Alignment and annotation | **rRNA** | 218 | 210 | 953 | 792 |
|  | **Functional categories** | 49,017 | 47,598 | 150,659 | 189,833 |
