# Supplementary material for: Microbial enrichment and gene functional categories revealed on the walls of a spent fuel pool of a nuclear power plant
Source: PLoS One. 2018 Oct 4;13(10):e0205228. doi: 10.1371/journal.pone.0205228 (PMC6171911; doi:10.1371/journal.pone.0205228)
Supplement: S3 Table — (DOCX) [file pone.0205228.s003.docx]

**S3 Table-**Alignment of ITS1 and ITS2 regions of close related Ustilaginomycetes Fungi:

CLUSTAL multiple sequence alignment

NR_145336_Moesziomyces_aphidis AGGTGTGGCTCGCACCTGTCTAACTAAA-TCGAGCTACCACATTTT----AACACGGTTG

EU427309_Sporisorium_scitamineum AGGTGTGGCTCGCACCTGTCTAACTAAACT-GGGCTACC--TATTT---CAACACGGTTG

AF135432_Sporisorium_reilianum AGGTGCGGCCCGCACCTGTCCAACTAAACTAGGGCTACT--TTTTT---CAACACGGTTG

NR_119765_Sporisorium_spinulosum AGGTGCGGCCCGCACCTGTCCAACTAAACTAGGGCTACC--TTTTT---CAACACGGTTG

AY740059_Sporisorium_manilense AGGTGTGGCCCGCACCTGTCCAACTAAACTAGGGCTACC-TTTTTT---CAACACGGTTG

AY345004_Ustilago_maydis AGGTGTGGCTCGCACCTGTCCAACTAAACCTGAGCTACC-TTTTTTATATAACACGGTTG

KF737866-Kalmanozyma_brasiliensis AGGTGTGGCTCGCACCTGTCCAACTAAACTTGAGCTACC---ATTT---GAACACGGTTG

NR_131991_Anthracocystis_themedae-arguentis AGGTGTGGCTCGCACCTGTCTAACTAAACTTGAGCTACC--TTTTT---CAACACGGTTG

AY344969_Anthracocystis_tumefaciens AGGTGTGGCTCGCACCTGTCTAACTAAACTTGAGCTACCTTTTTTT---CAACACGGTTG

AY345010_Ustilago_turcomanica AGGTGTGGCTCGCACCTGTCCAACTAAACTTGAGCTACC-TTTTTT---CAACACGGTTG

LT558136_Ustilago_bromivora AGGTGTGGCTCGCACCTGTCCAACTAAACTTGAGCTACC-TTTTTT---CAACACGGTTG

AF135424_Ustilago_tritici AGGTGTGGCTCGCACCTGTCCAACTAAACTTGAGCTACC-TTTTTT---CAACACGGTTG

NR_131997_Ustilago_hordei AGGTGTGGCTCGCACCTGTCCAACTAAACTTGAGCTACC--TTTTT---CAACACGGTTG

AF135430_Ustilago_nuda AGGTGTGGCTCGCACCTGTCCAACTAAACTTGAGCTACC--TTTTT---CAACACGGTTG

AY740062_Ustilago_avenae AGGTGTGGCTCGCACCTGTCCAACTAAACTTGAGCTACC--TTTTT---CAACACGGTTG

KF922222_Ustilago_sp._SMN03 AGGTGTGGCTCGCACCTGTCTAACTAAACTTGAGCTACC-TTTTTT---CAACACGGTTG

KP834590_Ustilago_cynodontis AGGTGTGGCTCGCACCTGTCTAACTAAACTTGAGCTACCATTTTTT---CAACACGGTTG

KY105788_Ustilago_shanxiensis AGGTGTGGCTCGCACCTGTCCAACTAAACTTGAGCTACC-TTTTTT---CAACACGGTTG

NR_132053_Ustilago_nunavutica AGGTGTGGCTCGCACCTGTCTAACTAAACTTGAGCTACC-TTTTTT---CAACACGGTTG

AY740065_Ustilago_calamagrostidis AGGTGTGGCTCGCACCTGTCTAACTAAACTTGAGCTACC-TTTTTT---CAACACGGTTG

SFP AGGTGTGGCTCGCACCTGTCTAACTAAAATTGAGCTACC--AATTT---CAACACGGTTG

FTC AGGTGTGGCTCGCACCTGTCTAACTAAAATTGAGCTACC--AATTT---CAACACGGTTG

AY740061_Ustilago_austro-africana AGGTGTGGCTCGCACCTGTCTAACTAAACTTGAGCTACC--TTTTT---CAACACGGTTG

NR_137546_Pseudozyma_hubeiensis AGGTGTGGCTCGCACCTGTCCAACTAAACTTGAGCTACC-TTTTTT---ATACACGGTTG

GQ167046_Ustilago_esculenta AGGTGTGGCTCGCACCTGTCTAAATAAACTTGAGCTACC-CAATTT---CAACACGGTTG

AY740169__Ustilago_davisii AGGTGTGGCTCGCACCTGTCTAACTAAACTTGAGCTACC-CAATTT---CAACACGGTTG

KF381026__Ustilago_filiformis AGGTGTGGCTCGCACCTGTCTAACTAAACTTGAGCTACC-CAATTT---CAACACGGTTG

***** *** ********** ** **** * ***** *** *********

NR_145336_Moesziomyces_aphidis CATCGGTT-GG------CTGTCAAACAGTGCG-----CGCGGCGATTTATTTCGCCTCCC

EU427309_Sporisorium_scitamineum CATCGGTTGGG-----TCTGCCAAACAGTG-------CACGAAAGTACCTGT--------

AF135432_Sporisorium_reilianum CATCGGTTGGG----TTGGGTCGAGCGAGTTTTCGGACTCGT------------------

NR_119765_Sporisorium_spinulosum CATCGGTTGGG-----TTTGTCGAGCGA--------------------------------

AY740059_Sporisorium_manilense CATCGGTTGGG---TCTTTGTCGAGCTGTGCGTGAG-CGCAGCGA---------------

AY345004_Ustilago_maydis CATCGGTC-GG-----TCTGTCGAAACCAGTAGCGCTCATAGCGAGCAGCGTCTGG----

KF737866-Kalmanozyma_brasiliensis CATCGGTTCGGAGAGAGATGTCCATTAGCGCGAGCTAGGCGGACGTGACCTCTT------

NR_131991_Anthracocystis_themedae-arguentis CATCGGTAGAG-------TGGCGAACG-------------------------------CG

AY344969_Anthracocystis_tumefaciens CATCGG-------------GTTGAGCGGTGAG----------------------------

AY345010_Ustilago_turcomanica CATCGGTC-GG-----CCTGTC-ACCAGTGCGACG--CAAGGAGAAAATCCTTGCGTCTG

LT558136_Ustilago_bromivora CATCGGTC-GG-----CCTGTCAAACAGTGCGACG--CAAGGAGAAAATCCTCGCGTCTG

AF135424_Ustilago_tritici CATCGGTC-GG-----CCTGTCAAACAGTGCGACG--CAAGGAGAAAATCCTCGCGTCTG

NR_131997_Ustilago_hordei CATCGGTC-GG-----CCTGTCAAACAGTGCGACG--CAAGGAGAAAATCCTCGCGTCTG

AF135430_Ustilago_nuda CATCGGTC-GG-----CCTGTCAAACAGCGCGACG--CAAGGAGAAAATCCTCGCGTCTG

AY740062_Ustilago_avenae CATCGGTC-GG-----CCTGTCAAACAGCGCGACG--CAAGGAGAAAATCCTCGCGTCTG

KF922222_Ustilago_sp._SMN03 CATCGGTT-GG-----CCTGTCAAACAGTGCGGCGGTCGCGAAATTGATTTT----TCGC

KP834590_Ustilago_cynodontis CATCGGTT-GG-----CCTGTCAATTAGTGCGGCGG-CGCGAATTTACTTTGCACCTGC-

KY105788_Ustilago_shanxiensis CATCGGTT-GG-----CCTGTCAAACAGTGCGGCGG-CGGAAATATTTTTTCTTTTTTCG

NR_132053_Ustilago_nunavutica CATCGGTT-GG-----CCTGTCAAACAGTGTG------GCGGCGTGAATTTTCACGTCTG

AY740065_Ustilago_calamagrostidis CATCGGTT-GG-----CCTGTCAAACAGTGCG------GCGGCGTGAATTTTCACGTCTG

SFP CATCGGTT-GG-----CCTGTCAAACAGTG-------CGCGGCG--------CGCTTCGG

FTC CATCGGTT-GG-----CCTGTCAAACAGTG-------CGCGGCG--------CGCTTCGG

AY740061_Ustilago_austro-africana CATCGGTTGGG-----CCTGTCAAGCGGCGTCGCT----------TTTTTTTTGAGCACG

NR_137546_Pseudozyma_hubeiensis CATCGGTC-GG-----CCTGTCCAACAGTGCG-----CGCGAAATTTATTTTC-------

GQ167046_Ustilago_esculenta CATCGGTT-AG------CTGTCAGACAGCGCG-----GACAGAGATTTTTTTTTTTTCTC

AY740169__Ustilago_davisii CATCGGTT-GG------CTGTCAAACAGTGC------CTCGGCGC---------------

KF381026__Ustilago_filiformis CATCGGTT-GG------CTGTCAAACAGTGC------CTCGGCGC---------------

****** *

NR_145336_Moesziomyces_aphidis CGCGCATTGCCGAGACGG----T-CGACA-TTTACCAA---AAACACTGTT-GATACCAT

EU427309_Sporisorium_scitamineum -------------GGAGGCAGCC-CGATAATCTACCA----AAACACTTTT-GATGATCT

AF135432_Sporisorium_reilianum -------------GAGGCCGGCC-TGACAACTTAACA----AAACACTTTT-GATGATCT

NR_119765_Sporisorium_spinulosum -GTTCTATACTTGCAAGGCGGCC-TGACAAATTATACCAAAAAACACTTTT-GATGATCT

AY740059_Sporisorium_manilense -------------GGCGGGGGCC-TGACACAACACC-----AAACACTTTT-GATGATCT

AY345004_Ustilago_maydis -----------GGAAAGACGGGT-CGGCGCTTCT-TAC---CAACACTTTT-GAACA-CT

KF737866-Kalmanozyma_brasiliensis ----------------------C-GGACACTTTA-CAC---AAACACTTTT-GATGATCT

NR_131991_Anthracocystis_themedae-arguentis CGTTCTTTGACGTGAGCTCGCCCTCTACACTTTA-CAC---AAACACTTTT-GATCTTCT

AY344969_Anthracocystis_tumefaciens CGTTCTCGGACGTGAAGCCC--T-CAACACTTTA-CAC---AAACACTTTT-GATTCTCT

AY345010_Ustilago_turcomanica C----------TGGGCGACGGAC-GGACAATTTTATT----TAACACTTTTGGATGATCT

LT558136_Ustilago_bromivora C----------TGGGCGACGGAC-GGACAATTTTATT----TAACACTTTTGGATGATCT

AF135424_Ustilago_tritici C----------TGGGCGACGGAC-GGACAATTTTATT----TAACACTTTTGGATGATCT

NR_131997_Ustilago_hordei C----------TGGGCGACGGAC-AGACAATTTTATT----GAACACTTTTTGATGATCT

AF135430_Ustilago_nuda C----------TGGGCGACGGAC-AGACAATTTTATT----GAACACTTTTTGATGATCT

AY740062_Ustilago_avenae C----------TGGGCGACGGAC-AGACAATTTTATT----GAACACTTTTTGATGATCT

KF922222_Ustilago_sp._SMN03 AGCTGCCCAACTCGGCGACGGAC-CGACACTTTTTACC---AAACACTTTT-GATGATCT

KP834590_Ustilago_cynodontis ------CAAGCTAGGCGACGGAC-CGACGCCTTACTTT---AAACACTTTT-GATGATCT

KY105788_Ustilago_shanxiensis CTTTGCCAAATTGGGCGACGGAC-CGACACTTAAT------CAACACTTTT-GATGATCT

NR_132053_Ustilago_nunavutica CT----TTGGCTGGGCGACGGAC-CGACACTTAAT------CAACACTTTT-GATGATCT

AY740065_Ustilago_calamagrostidis CT----TTGGCTGGGCGACGGAC-CGACACTTAAT------CAACACTTTT-GATGATCT

SFP CGCTCCGCTTCTGGGCGACGGCT--GACACATTATT-----AAACACTTTT-GATGATTT

FTC CGCTCCGCTTCTGGGCGACGGCT--GACACATTATT-----AAACACTTTT-GATGATTT

AY740061_Ustilago_austro-africana C----------TGGGAGACGGTCTCGACACTTTA-CAC---AAACACTTTT-GATGATCT

NR_137546_Pseudozyma_hubeiensis --CGCCGCGCTGAGCAGACGGGT-CGGCACTTTA-CAC---AGACACTTTT-GATAATCT

GQ167046_Ustilago_esculenta TGCTCGCTGCTGGGAAGGCGG-T-CGACACAATA-CAC---AAACACTTTT-GATGATCT

AY740169__Ustilago_davisii -----------TGGGAGGCGG-T-CGACACACAA-CAC---AAACACTTTT-GATGATCT

KF381026__Ustilago_filiformis -----------TGGGAGGCGG-T-CGACACACAA-CAC---AAACACTTTT-GATGATCT

***** ** ** *

NR_145336_Moesziomyces_aphidis AGG-ATTTGAACG----TAGATGA---------------AACTCGACTGGTAATGCGGTC

EU427309_Sporisorium_scitamineum AGG-ATTTGAAAG----TATTTAACATTT--------TA----CGACTGGTAATGCGGTC

AF135432_Sporisorium_reilianum AGGAATTTTGA------AAGTCTATCTTT-----------GAACGGCTGGTAATGCGGTC

NR_119765_Sporisorium_spinulosum AGGATTTTGAAAG----TATTTAACATTT------------TACGGCTGGTAATGCGGTC

AY740059_Sporisorium_manilense AGGATTTTGA-------AAGTTTACCTTT-----------TTACGGCTGGTAATGCGGTC

AY345004_Ustilago_maydis AGG-ATTGGAAGGACAAAAA-TCATTTTTTTGAT-GATGGAAGCGACTGGTAATGCGGTC

KF737866-Kalmanozyma_brasiliensis AGG-ATTTGAACG----AAGTTCAT-TTT---ATTGATGGAACCGACTGGTAATGCGGTC

NR_131991_Anthracocystis_themedae-arguentis AGG-ATTTGAATG---ACAATTTAT-------------------GACTGGTAATTCGGTC

AY344969_Anthracocystis_tumefaciens AGG-ATTGGAAGGA--------CAATTTT-------------ACGACTGGTAATTCGGTC

AY345010_Ustilago_turcomanica AGG-ATTTGAAGGAGAAAAAGTCATTTTT---ACGAATGAAATCGACTGGTAATGCGGTC

LT558136_Ustilago_bromivora AGG-ATTTGAAGGAGAAAAAGTCATTTTT---ACGAATGAAATCGACTGGTAATGCGGTC

AF135424_Ustilago_tritici AGG-ATTTGAAGGAGAAAAAGTCATTTTT---ACGAATGAAATCGACTGGTAATGCGGTC

NR_131997_Ustilago_hordei AGG-ATTTGAAGGAGAAAAAGTCATTTTT---ACAAATGAAATCGACTGGTAATGCGGTC

AF135430_Ustilago_nuda AGG-ATTTGAAGGAGAAAAAGTCATTTTT---ACGAATGAAATCGACTGGTAATGCGGTC

AY740062_Ustilago_avenae AGG-ATTTGAAGGAGAAAAAGTCATTTTT---ACGAATGAAATCGACTGGTAATGCGGTC

KF922222_Ustilago_sp._SMN03 AGG-ATTTGAATGAGAAAAGTTCATTTTT---ACAAATGAAATCGACTGGTAATGCGGTC

KP834590_Ustilago_cynodontis AGG-ATTTGAAGGATAAAAGTTCATTTTT---AC-AATGAAATCGACTGGTAATGCGGTC

KY105788_Ustilago_shanxiensis AGG-ATTTGAATGATAAAAGTTCATTTTT---ACAAATGAAATCGACTGGTAATGCGGTC

NR_132053_Ustilago_nunavutica AGG-ATTTGAATGATAAAAGTTCATTTTT---AC-AATGAAATCGACTGGTAATGCGGTC

AY740065_Ustilago_calamagrostidis AGG-ATTTGAATGATAAAAGTTCATTTTT---AC-AATGAAATCGACTGGTAATGCGGTC

SFP AGG-ATTTGAATG----AAGTTCATTTTT---AT-GATGGAACCGACTGGTAATGCGGTC

FTC AGG-ATTTGAATG----AAGTTCATTTTT---AT-GATGGAACCGACTGGTAATGCGGTC

AY740061_Ustilago_austro-africana AGG-ATTTGAATG----AAGTTCATTTTT---AC-GATGGAACCGACTGGTAATGCGGTC

NR_137546_Pseudozyma_hubeiensis AGG-ATTTGAATG---AAAGTTCATTTTT---AT-GATGGATCCGACTGGTAATGCGGTC

GQ167046_Ustilago_esculenta AGG-ATTTGAATG---AAAGTTCATTTTT---AT-GATGGAACCGACTGGTAATGCGGTC

AY740169__Ustilago_davisii AGGATTTTGAATG----AAGTTCATTTTT---AC-GATGGAACCGACTGGTAATGCGGTC

KF381026__Ustilago_filiformis AGGATTTTGAATG----AAGTTCATTTTT---AC-GATGGAACCGACTGGTAATGCGGTC

*** ** * * ******** *****

NR_145336_Moesziomyces_aphidis GTCTAAAATC--TAAAAACAACTTTTGGCAACGGATCTCTTGGTTCTCCCATCGATGAAG

EU427309_Sporisorium_scitamineum GTCTAAAATCT-AAAAAACAACTTTTGGCAACGGATCTCTTGGTTCTCCCATCGATGAAG

AF135432_Sporisorium_reilianum GTCTAAAAATG-GAAAAACAACTTTTGGCAACGGATCTCTTGGTTCTCCCATCGATGAAG

NR_119765_Sporisorium_spinulosum GTCTAAAATCTTAAAAAACAACTTTTGGCAACGGATCTCTTGGTTCTCCCATCGATGAAG

AY740059_Sporisorium_manilense GTCTAAAATTGTAAAAAACAACTTTTGGCAACGGATCTCTTGGTTCTCCCATCGATGAAG

AY345004_Ustilago_maydis GTCTAAATTG--AAAAAACAACTTTTGGCAACGGATCTCTTGGTTCTCCCATCGATGAAG

KF737866-Kalmanozyma_brasiliensis GTCTAAATCTA-TATATATAACTTTTGGCAACGGATCTCTTGGTTCTCCCATCGATGAAG

NR_131991_Anthracocystis_themedae-arguentis GTTTAAATTT---AAAAACAACTTTTGGCAACGGATCTCTTGGTTCTCCCATCGATGAAG

AY344969_Anthracocystis_tumefaciens GTTTAAATTT--TAAAAACAACTTTTGGCAACGGATCTCTTGGTTCTCCCATCGATGAAG

AY345010_Ustilago_turcomanica GTCTAATTTT---AAAAACAACTTTTGGCAACGGATCTCTTGGTTCTCCCATCGATGAAG

LT558136_Ustilago_bromivora GTCTAATTTT---AAAAACAACTTTTGGCAACGGATCTCTTGGTTCTCCCATCGATGAAG

AF135424_Ustilago_tritici GTCTAATTTT---AAAAACAACTTTTGGCAACGGATCTCTTGGTTCTCCCATCGATGAAG

NR_131997_Ustilago_hordei GTCTAATTTT---AAAAACAACTTTTGGCAACGGATCTCTTGGTTCTCCCATCGATGAAG

AF135430_Ustilago_nuda GTCTAATTTT---AAAAACAACTTTTGGCAACGGATCTCTTGGTTCTCCCATCGATGAAG

AY740062_Ustilago_avenae GTCTAATTTT---AAAAACAACTTTTGGCAACGGACCTCTTGGTTCTCCCATCGATGAAG

KF922222_Ustilago_sp._SMN03 GTCTAATTTT--TAAAAACAACTTTTGGCAACGGATCTCTTGGTTCTCCCATCGATGAAG

KP834590_Ustilago_cynodontis GTCTAATTTT---AAAAACAACTTTTGGCAACGGATCTCTTGGTTCTCCCATCGATGAAG

KY105788_Ustilago_shanxiensis GTCTAATTTT---AAAAACAACTTTTGGCAACGGATCTCTTGGTTCTCCCATCGATGAAG

NR_132053_Ustilago_nunavutica GTCTAATTTT--TAAAAACAACTTTTGGCAACGGATCTCTTGGTTCTCCCATCGATGAAG

AY740065_Ustilago_calamagrostidis GTCTAATTTT--TAAAAACAACTTTTGGCAACGGATCTCTTGGTTCTCCCATCGATGAAG

SFP GTCTAAATCT---AAAAACAACTTTTGGCAACGGATCTCTTGGTTCTCCCATCGATGAAG

FTC GTCTAAATCT---AAAAACAACTTTTGGCAACGGATCTCTTGGTTCTCCCATCGATGAAG

AY740061_Ustilago_austro-africana GACTAAATCT--AACAAACAACTTTTGGCAACGGATCTCTTGGTTCTCCCATCGATGAAG

NR_137546_Pseudozyma_hubeiensis GTCTAAATCT--AAAAAACAACTTTTGGCAACGGATCTCTTGGTTCTCCCATCGATGAAG

GQ167046_Ustilago_esculenta GTCTAAATCT--AAAAAACAACTTTTGGCAACGGATCTCTTGGTTCTCCCATCGATGAAG

AY740169__Ustilago_davisii GTCTAAATCT--AAAAAACAACTTTTGGCAACGGATCTCTTGGTTCTCCCATCGATGAAG

KF381026__Ustilago_filiformis GTCTAAATCT--AAAAAACAACTTTTGGCAACGGATCTCTTGGTTCTCCCATCGATGAAG

* *** * * * **************** ************************

NR_145336_Moesziomyces_aphidis AACGCAGCGAATTGCGATAAGTAATGTGAATTGCAGAAGTGAATCATCGAATCTTTGAAC

EU427309_Sporisorium_scitamineum AACGCAGCGAATTGCGATAAGTAATGTGAATTGCAGAAGTGAATCATCGAATCTTTGAAC

AF135432_Sporisorium_reilianum AACGCAGCGAATTGCGATAAGTAATGTGAATTGCAGAAGTGAATCATCGAATCTTTGAAC

NR_119765_Sporisorium_spinulosum AACGCAGCGAATTGCGATAAGTAATGTGAATTGCAGAAGTGAATCATCGAATCTTTGAAC

AY740059_Sporisorium_manilense AACGCAGCGAATTGCGATAAGTAATGTGAATTGCAGAAGTGAATCATCGAATCTTTGAAC

AY345004_Ustilago_maydis AACGCAGCGAATTGCGATAAGTAATGTGAATTGCAGAAGTGAATCATCGAATCTTTGAAC

KF737866-Kalmanozyma_brasiliensis AACGCAGCGAATTGCGATAAGTAATGTGAATTGCAGAAGTGAATCATCGAATCTTTGAAC

NR_131991_Anthracocystis_themedae-arguentis AACGCAGCGAATTGCGATAAGTAATGTGAATTGCAGAAGTGAATCATCGAATCTTTGAAC

AY344969_Anthracocystis_tumefaciens AACGCAGCGAATTGCGATAAGTAATGTGAATTGCAGAAGTGAATCATCGAATCTTTGAAC

AY345010_Ustilago_turcomanica AACGCAGCGAATTGCGATAAGTAATGTGAATTGCAGAAGTGAATCATCGAATCTTTGAAC

LT558136_Ustilago_bromivora AACGCAGCGAATTGCGATAAGTAATGTGAATTGCAGAAGTGAATCATCGAATCTTTGAAC

AF135424_Ustilago_tritici AACGCAGCGAATTGCGATAAGTAATGTGAATTGCAGAAGTGAATCATCGAATCTTTGAAC

NR_131997_Ustilago_hordei AACGCAGCGAATTGCGATAAGTAATGTGAATTGCAGAAGTGAATCATCGAATCTTTGAAC

AF135430_Ustilago_nuda AACGCAGCGAATTGCGATAAGTAATGTGAATTGCAGAAGTGAATCATCGAATCTTTGAAC

AY740062_Ustilago_avenae AACGCAGCGAATTGCGATAAGTAATGTGAATTGCAGAAGTGAATCATCGAATCTTTGAAC

KF922222_Ustilago_sp._SMN03 AACGCAGCGAATTGCGATAAGTAATGTGAATTGCAGAAGTGAATCATCGAATCTTTGAAC

KP834590_Ustilago_cynodontis AACGCAGCGAATTGCGATAAGTAATGTGAATTGCAGAAGTGAATCATCGAATCTTTGAAC

KY105788_Ustilago_shanxiensis AACGCAGCGAATTGCGATAAGTAATGTGAATTGCAGAAGTGAATCATCGAATCTTTGAAC

NR_132053_Ustilago_nunavutica AACGCAGCGAATTGCGATAAGTAATGTGAATTGCAGAAGTGAATCATCGAATCTTTGAAC

AY740065_Ustilago_calamagrostidis AACGCAGCGAATTGCGATAAGTAATGTGAATTGCAGAAGTGAATCATCGAATCTTTGAAC

SFP AACGCAGCGAATTGCGATAAGTAATGTGAATTGCAGAAGTGAATCATCGAATCTTTGAAC

FTC AACGCAGCGAATTGCGATAAGTAATGTGAATTGCAGAAGTGAATCATCGAATCTTTGAAC

AY740061_Ustilago_austro-africana AACGCAGCGAATTGCGATAAGTAATGTGAATTGCAGAAGTGAATCATCGAATCTTTGAAC

NR_137546_Pseudozyma_hubeiensis AACGCAGCGAATTGCGATAAGTAATGTGAATTGCAGAAGTGAATCATCGAATCTTTGAAC

GQ167046_Ustilago_esculenta AACGCAGCGAATTGCGATAAGTAATGTGAATTGCAGAAGTGAATCATCGAATCTTTGAAC

AY740169__Ustilago_davisii AACGCAGCGAATTGCGATAAGTAATGTGAATTGCAGAAGTGAATCATCGAATCTTTGAAC

KF381026__Ustilago_filiformis AACGCAGCGAATTGCGATAAGTAATGTGAATTGCAGAAGTGAATCATCGAATCTTTGAAC

************************************************************

NR_145336_Moesziomyces_aphidis GCACCTTGCGCTCCCGGCAGATCTAATCTGGGGAGCATGCCTGTTTGAGGGCCGCGAATT

EU427309_Sporisorium_scitamineum GCACCTTGCGCTCCTTGCAGATCTAATCTGGGGAGCATGCCTATTTGAGGGCCGCGAATT

AF135432_Sporisorium_reilianum GCACCTTGCGCTCCCTGCAGATCTAATCTGGGGAGCATGCCTGTTTGAGGGCCGCGAATT

NR_119765_Sporisorium_spinulosum GCACCTTGCGCTCCCTGCAGATCTAATCTGGGGAGCATGCCTGTTTGAGGGCCGCGAATT

AY740059_Sporisorium_manilense GCACCTTGCGCTCCCTGCAGATCTAATCTGGGGAGCATGCCTGTTTGAGGGCCGCGAATT

AY345004_Ustilago_maydis GCACCTTGCGCTCCCGGCAGATTTAATCTGGGGAGCATGCCTGTTTGAGGGCCGCGAATT

KF737866-Kalmanozyma_brasiliensis GCACCTTGCGCTCCCGGCAGATCTAATCTGGGGAGCATGCCTGTTTGAGGGCCGCGAATT

NR_131991_Anthracocystis_themedae-arguentis GCACCTTGCGCTCCCTGCAGATCTAATCTGGGGAGCATGCCTGTTTGAGGGCCGCGAATT

AY344969_Anthracocystis_tumefaciens GCACCTTGCGCTCCCTGCAGATCTAATCTGGGGAGCATGCCTGTTTGAGGGCCGCGAATT

AY345010_Ustilago_turcomanica GCACCTTGCGCTCCCGGCAGATCTAATCTGGGGAGCATGCCTGTTTGAGGGCCGCGAATT

LT558136_Ustilago_bromivora GCACCTTGCGCTCCCGGCAGATCTAATCTGGGGAGCATGCCTGTTTGAGGGCCGCGAATT

AF135424_Ustilago_tritici GCACCTTGCGCTCCCGGCAGATCTAATCTGGGGAGCATGCCTGTTTGAGGGCCGCGAATT

NR_131997_Ustilago_hordei GCACCTTGCGCTCCCGGCAGATCTAATCTGGGGAGCATGCCTGTTTGAGGGCCGCGAATT

AF135430_Ustilago_nuda GCACCTTGCGCTCCCGGCAGATCTAATCTGGGGAGCATGCCTGTTTGAGGGCCGCGAATT

AY740062_Ustilago_avenae GCACCTTGCGCTCCCGGCAGATCTAATCTGGGGAGCATGCCTGTTTGAGGGCCGCGAATT

KF922222_Ustilago_sp._SMN03 GCACCTTGCGCTCCCGGCAGATCTAATCTGGGGAGCATGCCTGTTTGAGGGCCGCGAATT

KP834590_Ustilago_cynodontis GCACCTTGCGCTCCCGGCAGATCTAATCTGGGGAGCATGCCTGTTTGAGGGCCGCGAATT

KY105788_Ustilago_shanxiensis GCACCTTGCGCTCCCTGCAGATCTAATCTGGGGAGCATGCCTGTTTGAGGGCCGCGAATT

NR_132053_Ustilago_nunavutica GCACCTTGCGCTCCCGGCAGATCTAATCTGGGGAGCATGCCTGTTTGAGGGCCGCGAATT

AY740065_Ustilago_calamagrostidis GCACCTTGCGCTCCCGGCAGATCTAATCTGGGGAGCATGCCTGTTTGAGGGCCGCGAATT

SFP GCACCTTGCGCTCCCGGCAGATCTAATCTGGGGAGCATGCCTGTTTGAGGGCCGCGAATT

FTC GCACCTTGCGCTCCCGGCAGATCTAATCTGGGGAGCATGCCTGTTTGAGGGCCGCGAATT

AY740061_Ustilago_austro-africana GCACCTTGCGCTCCCTGCAGATCTAATCTGGGGAGCATGCCTGTTTGAGGGCCGCGAATT

NR_137546_Pseudozyma_hubeiensis GCACCTTGCGCTCCCGGCAGATCTAATCTGGGGAGCATGCCTGTTTGAGGGCCGCGAATT

GQ167046_Ustilago_esculenta GCACCTTGCGCTCCCGGCAGATCTAATCTGGGGAGCATGCCTGTTTGAGGGCCGCGAATT

AY740169__Ustilago_davisii GCACCTTGCGCTCCCGGCAGATCTAATCTGGGGAGCATGCCTGTTTGAGGGCCGCGAATT

KF381026__Ustilago_filiformis GCACCTTGCGCTCCCGGCAGATCTAATCTGGGGAGCATGCCTGTTTGAGGGCCGCGAATT

************** ****** ******************* *****************

NR_145336_Moesziomyces_aphidis GTTTCGAACGACAGCTTTCTTATTTAGTT--GAGAAAGCTGGC-GGATCGGTATTGAGGG

EU427309_Sporisorium_scitamineum GTTTCGAACGCATGCTTTTTTTATTAC----GAAAGAGCTGGC-GGATCGGTAGTGAGGG

AF135432_Sporisorium_reilianum GTTTCGAACCCACGCTTTTTTAAGT--------AAAGGCGTGCGGGATCGGTAGTGAGGG

NR_119765_Sporisorium_spinulosum GTTTCGAGCGCACGCTTTTTTCAGT--------AAAGGCATGC-GGATCGGTAGTGAGGG

AY740059_Sporisorium_manilense GTTTCGAACCCACGCTTTTTTCAGT--------AAAGGCGTGC-GGATCGGTAGTGAGGG

AY345004_Ustilago_maydis GTTTCGAACGACAGCTTTTTTTTCTTGTTGAGAAAGAGCTGGC-GGATCGGTAGTGAGGG

KF737866-Kalmanozyma_brasiliensis GTTTCGAACGGCAGCTTTTTTCACG--------AAGAGCTGGC-GGATCGGTTTTGAGGG

NR_131991_Anthracocystis_themedae-arguentis GTTTCGAACCATC-CTTTTTTTATATATAGAAAAAGGGTT----GGATCGGTAGTGAGGG

AY344969_Anthracocystis_tumefaciens GTTTCGAGCCGACCATTTTTTTTTAAATT----AAAAGTGGAC-GGATCGGTAATGAGGG

AY345010_Ustilago_turcomanica GTTTCGAACGACAGCTTTTTTCTTTTG----GAAAAGGTTGAC-GGATCGGTATTGAGGG

LT558136_Ustilago_bromivora GTTTCGAACGACAGCTTTTTTCTTTTG----GAAAAGGTTGAT-GGATCGGTATTGAGGG

AF135424_Ustilago_tritici GTTTCGAACGACAGCTTTTTTCTTTTG----GAAAAGGTTGAC-GGATCGGTATTGAGGG

NR_131997_Ustilago_hordei GTTTCGAACGACAGCTTTTTTCTTTTG----GAAAAGGTTGAC-GGATCGGTATTGAGGG

AF135430_Ustilago_nuda GTTTCGAACGACAGCTTTTTTCTTTTG----GAAAAGGTTGAC-GGATCGGTATTGAGGG

AY740062_Ustilago_avenae GTTTCGAACGACAGCTTTTTTCTTTTG----GAAAAGGTTGAC-GGATCGGTATTGAGGG

KF922222_Ustilago_sp._SMN03 GTTTCGAACGACGACTTTTTTCA--------CAAAGAGTTGGC-GGATCGGTGTTGAGAG

KP834590_Ustilago_cynodontis GTTTCGAACGACAACTTTTTTCA--------CAAAGAGTTGGC-GGATCGGTATTGAGGG

KY105788_Ustilago_shanxiensis GTTTCGAACGACAGCTTTTTTCACG--------AAAAGTTGGC-GGATCGGTATTGAGGG

NR_132053_Ustilago_nunavutica GTTTCGAACGACAACTTTTTTCTTTTG------AAGAGTTGGC-GGATCGGTATTGAGGG

AY740065_Ustilago_calamagrostidis GTTTCGAACGACAACTTTTTTTTTTG-------AAGAGTTGGC-GGATCGGTATTGAGGG

SFP GTTTCGAACGGCAGCTTTCTTTTAC------GAGAAGGCTGGC-GGATCGGTAGTGAGGA

FTC GTTTCGAACGGCAGCTTTCTTTTAC------GAGAAGGCTGGC-GGATCGGTAGTGAGGA

AY740061_Ustilago_austro-africana GTTTCGAACGGCAGCTTTTTTCACG--------AAAAGTTGGC-GGATCGGTAGTGAGGA

NR_137546_Pseudozyma_hubeiensis GTTTCGAACGACAGCTTTTTTCAGT--------AAGAGCTGGC-GGATCGGTATTGAGGG

GQ167046_Ustilago_esculenta GTTTCGAACGACAGCTTTCTTCTTTGC----AAGAGAGTTGGC-GGATCGGTAATGAGGG

AY740169__Ustilago_davisii GTTTCGAACGACAGCTTTTTTCTTTGT----GAAAAAGTTGGC-GGATCGGTATTGAGGG

KF381026__Ustilago_filiformis GTTTCGAACGACAGCTTTTTTTCTTTGT---GAAAAAGTTGGC-GGATCGGTATTGAGGG

******* * *** ** * * ******** ****

NR_145336_Moesziomyces_aphidis -----TCTTGCCATCTTCCACGGTGGCTCCCTCGAAATGCATTAGCGCATCCA-TTCGAT

EU427309_Sporisorium_scitamineum -----TTTTGCCA-TT--TACCGTGGCTCCCTCGAAATGCATTAGTGCATCCA-TTTGAC

AF135432_Sporisorium_reilianum -------TTGCCA-TT--CACCGTGGCTCGCTCGAAATGCATTAGCGCATCCA-TTGAAT

NR_119765_Sporisorium_spinulosum ----TTTTTGCCA-TT--AACCGTGGCTCCCTCGAAATGCATTAGTGCATCCA-TTTAAT

AY740059_Sporisorium_manilense --TTTTTTTGCCATTT--CACCGTGGCTCCCTCGAAATGCATTAGTGCATCCA-TTTGAT

AY345004_Ustilago_maydis ----TCTCTGCCA-TT--TACCGTGGCTCCCTCGAAATGCATTAGCGCATCCA--TTGGA

KF737866-Kalmanozyma_brasiliensis ---TCTTTTGCCA-TT--TACCGTGGCTCCCTCTAAATGCATTAGCGCATCCA-TTTGAT

NR_131991_Anthracocystis_themedae-arguentis ------TTTGCCA-TT--CACCGTGGCTCCCTCGAAATACATTAGCGCATCCA-TTTGAT

AY344969_Anthracocystis_tumefaciens GTTTTTTTTGCCA-TT--CACCGTGGCTCCCTCGAAATACATTAGCGCATCCA-TTTGAT

AY345010_Ustilago_turcomanica ----TTTTTGCCA-TT--TACTGTGGCTCCCTCGAAATAGATTAGCGCATCCA-TTTTAT

LT558136_Ustilago_bromivora ----TTTTTGCCA-TT--TACCGTGGCTCCCTCGAAATAGATTAGCGCATCCA-TTTTAT

AF135424_Ustilago_tritici ----TTTTTGCCA-TT--TACCGTGGCTCCCTCGAAATAGATTAGCGCATCCA-TTTTAT

NR_131997_Ustilago_hordei ----TTTTTGCCA-TT--TACCGTGGCTCCCTTGAAATAGATTAGCGCATCCA-TTTTAT

AF135430_Ustilago_nuda ----TTTTTGCCA-TT--TATCGTGGCTCCCTTGAAATAGATTAGCGCATCCA-TTTTAT

AY740062_Ustilago_avenae ----TTTTTGCCA-TT--TACCGTGGCTCCCTTGAAATAGATTAGC--------TTTTAT

KF922222_Ustilago_sp._SMN03 ---TTTTTTGCCA-TT--CACCGTGGCTCTCTCGAAATGCATTAGCGCATCCA-TTTGAT

KP834590_Ustilago_cynodontis -----TTTTGCCA-TT--CACCGTGGCTCTCTCGAAATGCATTAGCGCATCCA-TTTGAT

KY105788_Ustilago_shanxiensis -----TTTTGCCA-TT--CACCGTGGCTCCCTCGAAATGCATTAGCGCATCCA-TTTGAT

NR_132053_Ustilago_nunavutica -----TTTTGCCA-TT--CACCGTGGCTCCCTCGAAATGCATTAGCGCATCCA-TTTGAT

AY740065_Ustilago_calamagrostidis -----TTTTGCCA-TT--CACCGTGGCTCCCTCGAAATGCATTAGCGCATCCA-TTTGAT

SFP -------TTGCCA-TTAACACCATGGCTTCCTCGAAAAGCATTAGCGCATCCA-TTTGAT

FTC -------TTGCCA-TTAACACCATGGCTTCCTCGAAAAGCATTAGCGCATCCA-TTTGAT

AY740061_Ustilago_austro-africana TTTTTTTTTGCCA-TT--CACCGTGGCTTCCTCGAAAAGCATTAGCGCATCCA-TTTGAT

NR_137546_Pseudozyma_hubeiensis ----TCTTTGCCA-TT--TACCGTGGCTCCCTCGAAATGCATTAGCGCATCCA-TTTGAT

GQ167046_Ustilago_esculenta ----TTTTTGCCA-TT--TACCGTGGCTCCCTCGAAATGCATTAGCGCATCCA-TTCAAT

AY740169__Ustilago_davisii ------TTTGCCA-TT--TACCATGGCTCCCTCGAAATGCATTAGCGCATCCA-TTTGAT

KF381026__Ustilago_filiformis ------TTTGCCA-TT--TACCATGGCTCCCTCGAAATGCATTAGCGCATCCA-TTTGAT

***** * * ***** ** *** ***** *

NR_145336_Moesziomyces_aphidis --AGGC--AAG-ACGGACGAAAGCTCGTTATTT--CGCCCACGTCTTTCCCTGCCGGGTT

EU427309_Sporisorium_scitamineum --AGGC--AAAGACGGACGAAGGCTCGACTTTT--GGCCCA--TC-TTCCCTGCCAGGTT

AF135432_Sporisorium_reilianum TGAGGC--AAAGACGGACGAAGGCTCGCC-TTT--CGCTCTC-TC-TTCCTTGCCGGGTT

NR_119765_Sporisorium_spinulosum --AGGC--AAAGACGGACGAAAGCTCATCTTTT--CGCTCTT-TC-TTCCCTGCCGGGTT

AY740059_Sporisorium_manilense --AGGC--AAAGACGGACGAAGGCTCGAC-TTT--CGCTCCC-TC-TTCCTTGCCGGGTT

AY345004_Ustilago_maydis --AGGCGGAAAGACGGACGAAAGCTCGAGTTTTTTTGCCCTC-GC-TTCCCTGCCGGGTT

KF737866-Kalmanozyma_brasiliensis --AGGC--AAG-ACGGACGAAAGCTCAATCTTT--CGCTCTTTTC-TTTCCTGCCGGGTT

NR_131991_Anthracocystis_themedae-arguentis --AGGC--AAGAACGGACGAAAGCTCATC-TTT--CGCTCTC-TC-TTCCCTGCCGGGTT

AY344969_Anthracocystis_tumefaciens --AGGC--AAGAACGGACGAAAGCTCACTTTTT--CGCCCTTTTTCTTCCCTGCCGGGTT

AY345010_Ustilago_turcomanica --AGGC--AAG-ACGGACGAAAGCTCGAT-TTT--TGCTCTC-TC-TTCCCTGCCGGGTT

LT558136_Ustilago_bromivora --AGGC--AAG-ACGGACGAAAGCTCGAT-TTT--TGCTCTC-TC-TTCCCTGCCGGGTT

AF135424_Ustilago_tritici --AGGC--AAG-ACGGACGAAAGCTCGAT-TTT--TGCTCTC-TC-TTCCCTGCCGGGTT

NR_131997_Ustilago_hordei --AGGC--AAG-ACGGACGAAAGCTCGAT-TTT--TGCTCTC-TC-TTCCCTGCCGGGTT

AF135430_Ustilago_nuda --AGGC--AAG-ACGGACGAAAGCTCGAT-TTT--TGCTCTC-TC-TTCCCTGCCGGGTT

AY740062_Ustilago_avenae --AGGC--AAG-ACGGACGAAAGCTCGAT-TTT--TGCTCTC-TC-TTCCCTGCCGGGTT

KF922222_Ustilago_sp._SMN03 --AGGC--AAG-ACGGACGAAAGCTCATCTTTT--CGCTCTC-TC-TTCCCTGCCGGGTT

KP834590_Ustilago_cynodontis --AGGC--AAG-ACGGACGAAAGCTCCTC-TTT--CGCTCTC-TC-TTCCCTGCCGGGTT

KY105788_Ustilago_shanxiensis --AGGC--AAG-ACGGACGAAAGCTCATC-TTT--CGCTCTC-TC-TTCCCTGCCGGGTT

NR_132053_Ustilago_nunavutica --AGGC--AAG-ACGGACGAAAGCTCGAT-TTT--CGCTCTC-TC-TTCCCTGCCGGGTT

AY740065_Ustilago_calamagrostidis --AGGC--AAG-ACGGACGAAAGCTCAAT-TTT--CGCTCTC-TC-TTCCCTGCCGGGTT

SFP --AGGC--AAG-ACGGACGAAAGCTTGTA-TTT--CGCCTTC--C-TTCCCTGCCGGGTT

FTC --AGGC--AAG-ACGGACGAAAGCTTGTA-TTT--CGCCTTC--C-TTCCCTGCCGGGTT

AY740061_Ustilago_austro-africana --AGGC--AAG-ACGGACGAAAGCTCGAATTTT--CGCCCTCATC-TTCCCTGCCGGGTT

NR_137546_Pseudozyma_hubeiensis --AGGCGAAAG-ACGGACGAAAGCTCGAT-TTT--CGCCCTC-TC-TTCCCTGCCGGGTT

GQ167046_Ustilago_esculenta --AGGC--AAG-ACGGACGAAAGCTTGAA-TTT--CGCCCTC-TC-TTTCCTGCCGGGTT

AY740169__Ustilago_davisii --AGGC--AAG-ACGGACGAAAGCTCGAT-TTT--CGCCCTC-TC-TTCCCTGCCGGGTT

KF381026__Ustilago_filiformis --AGGC--AAG-ACGGACGAAAGCTCGAT-TTT--CGCCCTC-TC-TTCCCTGCCGGGTT

**** ** ********* *** *** ** ** * **** ****

NR_145336_Moesziomyces_aphidis TTGATAATATCAGGACTTCGGAGAGGAGAGGCGCAGGGTCGAGGAGCTGGACGCGACG--

EU427309_Sporisorium_scitamineum TTGATAATATCAGGACTTTGGTGGTG-AGG----ATGAGCAAGAAGCTGGACGCGACGGC

AF135432_Sporisorium_reilianum TTGATACTATCAGGGCTTCGGAGGCG-GAG--AGA-GGGCA-TGAGCTGGACGCGACGAC

NR_119765_Sporisorium_spinulosum TTGATAATATCAGGACTTCGGAGGCG-GAGAAAAA-AAGCA-AGAGCTGGACGCAACGAC

AY740059_Sporisorium_manilense TTGATACTATCAGGACTTCGGAAGCG-GAG--AGA-GGGCA-AGAGCTGGACGCGACGGC

AY345004_Ustilago_maydis TTGATAATGTCAGGACTTCGGAGGCG-AAG--AGA-GGGCG-GGAGCTGGACGCAACGAC

KF737866-Kalmanozyma_brasiliensis TTGATAATATCGAAGCTTCGGAGAGA-CTG--AAAAGAGCAACGAGCTGGACGCAACG--

NR_131991_Anthracocystis_themedae-arguentis TTGATAATATCAGGACTTCGGAGGCG-GAG--AAA-GGGCA-AGAGCTGGACGCAACGA-

AY344969_Anthracocystis_tumefaciens TTGATAATATCAGGACTTCGGAGGCG-GAG--AAA-GGGTA-CGAGCTGGACGCAACG-T

AY345010_Ustilago_turcomanica TTGATACTATCAGGACTTCGGAGAGG-TTG--AGATGGGTA-GGAGCTGGACGCAACG--

LT558136_Ustilago_bromivora TTGATACTATCAGGACTTCGGAGAGG-TTG--AGATGGGTA-GGAGCTGGACGCAACG--

AF135424_Ustilago_tritici TTGATACTATCAGGACTTCGGAGAGG-TTG--AGATGGGTA-GGAGCTGGACGCAACG--

NR_131997_Ustilago_hordei TTGATACTATCAGGACTTCGGAGAGG-TTG--AGATGGGTA-GGAGCTCGACGCAACG--

AF135430_Ustilago_nuda TTGATACTATCAGGACTTCGGAGAGG-TTG--AGATGGGTA-GGAGCTCGACGCAACG--

AY740062_Ustilago_avenae TTGATACTATCAGGACTTCGGAGAGG-TTG--AGATGGGTA-GGAGCTCGACGCAACG--

KF922222_Ustilago_sp._SMN03 TTGATAATATCAGGACTTCGGAGAGG-TTG--AGATGGGTA-CGAGCTGGACGCAACG--

KP834590_Ustilago_cynodontis TTGATAATATCAGGACTTCGGAGAGG-TTG--AGATGGGTACGGAGCTGGACGCAACG--

KY105788_Ustilago_shanxiensis TTGATAATATCAGGACTTCGGAGAGG-TTG--AGATGGGTA-AGAGCTGGACGCAACG--

NR_132053_Ustilago_nunavutica TTGATAATATCAGGACTTCGGAGAGG-TTG--AGATGGGTA-AGAGCTGGACGCAACG--

AY740065_Ustilago_calamagrostidis TTGATAATATCAGGACTTCGGAGAGG-TTG--AGATGGGTA-AGAGCTGGACGCAACG--

SFP TTGATAATATCAGGACTTCGGAGGCG-AAG--GGA--GGTTTCGAGCTGGACGCAACG--

FTC TTGATAATATCAGGACTTCGGAGGCG-AAG--GGA--GGTTTCGAGCTGGACGCAACG--

AY740061_Ustilago_austro-africana TTGATAATATCAGGACTTCGGAGGCG-GAG--AGA-GGGTT-CAAGCTGGACGCAACG--

NR_137546_Pseudozyma_hubeiensis TTGATAATATCAGGACTTCGGAGGCG-GAG--AAA-GGGTT-AGAGCTGGACGCAACGAC

GQ167046_Ustilago_esculenta TTGATAATATCAGGGCTTCGGAGGCA-GAG--AGATGGGTT-AGAGCTGGACGCAACG--

AY740169__Ustilago_davisii TTGATAATATCAGGACTTCGGAGGCG-GAG--AAA-GGGTT-AGAGCTGGACGCAACG--

KF381026__Ustilago_filiformis TTGATAATATCAGGACTTCGGAGGCG-GAG--AAA-GGGTT-AGAGCTGGACGCAACG--

****** * ** *** ** * * **** ***** ***

NR_145336_Moesziomyces_aphidis TTT--TGCTGGTTGGAGTGCTTCTGAACCCCGCC

EU427309_Sporisorium_scitamineum CTT--TGCTGATTGGAGTGCTTCTGAACACCGCC

AF135432_Sporisorium_reilianum TTT--GTCGGTTGGAAGTGCTTCTGAACCCCGCC

NR_119765_Sporisorium_spinulosum TTT--TGCTGGTTGGAGTGCTTCTGAACCCCGCC

AY740059_Sporisorium_manilense ATT---GCTGTTTGGAGTGCTTCTGAACCCCGCC

AY345004_Ustilago_maydis TTTTCTGCTGTTTGGCGTGCTTCTGAACCCCGCC

KF737866-Kalmanozyma_brasiliensis ACT--TGCTGGTTGGAGCGCTTCTGAACCCCGCC

NR_131991_Anthracocystis_themedae-arguentis TCT--TGCTGTTTGGCGTGCTTCTGAACCCCGCC

AY344969_Anthracocystis_tumefaciens TTT--TGCTGTTTGGCGTGCTTCTGAACCCCGCC

AY345010_Ustilago_turcomanica GCT--TGCTGTTTGGAGTGCTTCTGAAACCCGCC

LT558136_Ustilago_bromivora GCT--TGCTGTTTGGAGTGCTTCTGAAACCCGCC

AF135424_Ustilago_tritici GCT--TGCTGTTTGGAGTGCTTCTGAAACCCGCC

NR_131997_Ustilago_hordei GCT--TGCTGTTTGGAGTGCTTCTGAAACCCGCC

AF135430_Ustilago_nuda GCT--TGCTGTTTGGAGTGCTTCTGAAACCCGCC

AY740062_Ustilago_avenae GCT--TGCTGTTTGGAGTGCTTCTGAAACCCGCC

KF922222_Ustilago_sp._SMN03 ACT--TGCTGTTTGGAGTGCTTCTGAAACCCGCC

KP834590_Ustilago_cynodontis GCT--TGCTGTTTGGAGTGCTTCTGAAACCCGCC

KY105788_Ustilago_shanxiensis GCT--TGCTGTTTGGAGTGCTTCTGAAACCCGCC

NR_132053_Ustilago_nunavutica GCT--TGCTGTTTGGAGTGCTTCTGAAACCCGCC

AY740065_Ustilago_calamagrostidis GCT--TGCTGTTTGGAGTGCTTCTGAAACCCGCC

SFP TCT--TGCTGGTTGGAGTGCTTCT-AACCCCGCC

FTC TCT--TGCTGGTTGGAGTGCTTCT-AACCCCGCC

AY740061_Ustilago_austro-africana TTT--TGCTGGTTGGAGTGCTTCT-AACCCCGCC

NR_137546_Pseudozyma_hubeiensis TTT--TGCTGGTTGGAGTGCTTCTGAACCCCGCC

GQ167046_Ustilago_esculenta TTT--TGCTGGTTGGAGTGCTTCTGAAACCCGCC

AY740169__Ustilago_davisii TTT--TGCTGTTTGGAGTGCTTCTGAAACCCGCC

KF381026__Ustilago_filiformis TTT--TGCTGTTTGGAGTGCTTCTGAAACCCGCC

* * * * * * ****** ** *****
