# Supplementary material for: Microbial enrichment and gene functional categories revealed on the walls of a spent fuel pool of a nuclear power plant
Source: PLoS One. 2018 Oct 4;13(10):e0205228. doi: 10.1371/journal.pone.0205228 (PMC6171911; doi:10.1371/journal.pone.0205228)
Supplement: S4 Table — (DOCX) [file pone.0205228.s004.docx]

**S4 Table:** Gene function associated with respiration found in the SFP sample.

|  |
| --- |
| [NiFe] hydrogenase metallocenter assembly protein HypD |
| AA3-600 quinol oxidase subunit I |
| AA3-600 quinol oxidase subunit II |
| ATP synthase A chain (EC 3.6.3.14) |
| ATP synthase alpha chain (EC 3.6.3.14) |
| ATP synthase beta chain (EC 3.6.3.14) |
| ATP synthase C chain (EC 3.6.3.14) |
| Butyryl-CoA dehydrogenase (EC 1.3.99.2) |
| Ccs1/ResB-related putative cytochrome C-type biogenesis protein |
| cytochrome b-5 |
| Cytochrome C heme lyase CCHL (EC 4.4.1.17) |
| Cytochrome c oxidase polypeptide I (EC 1.9.3.1) |
| Cytochrome c oxidase polypeptide II (EC 1.9.3.1) |
| Cytochrome c oxidase polypeptide III (EC 1.9.3.1) |
| Cytochrome c oxidase polypeptide Va, mitochondrial precursor (EC 1.9.3.1) |
| Cytochrome c oxidase polypeptide Vb, mitochondrial precursor (EC 1.9.3.1) |
| Cytochrome c-type biogenesis protein CcmE, heme chaperone |
| Cytochrome C1 heme lyase CCHL (EC 4.4.1.17) |
| Cytochrome c551/c552 |
| Cytochrome O ubiquinol oxidase subunit I (EC 1.10.3.-) |
| Cytochrome oxidase biogenesis protein Cox11-CtaG, copper delivery to Cox1 |
| Cytochrome oxidase biogenesis protein Mss51, required for maturation, translation of COX1 mRNA |
| Cytochrome oxidase biogenesis protein Sco1/SenC/PrrC, putative copper metallochaperone |
| D-amino acid dehydrogenase small subunit (EC 1.4.99.1) |
| Electron transfer flavoprotein-ubiquinone oxidoreductase (EC 1.5.5.1) |
| Ferredoxin |
| Ferredoxin--NADP(+) reductase (EC 1.18.1.2) |
| Ferredoxin, 2Fe-2S |
| Glycerol dehydrogenase (EC 1.1.1.6) |
| Glycerol-3-phosphate dehydrogenase (EC 1.1.5.3) |
| Heme A synthase, cytochrome oxidase biogenesis protein Cox15-CtaA |
| Heme O synthase, protoheme IX farnesyltransferase (EC 2.5.1.-) COX10-CtaB |
| L-lactate dehydrogenase (EC 1.1.2.3) |
| NAD-dependent formate dehydrogenase (EC 1.2.1.2) |
| NADH dehydrogenase (EC 1.6.99.3) |
| NADH dehydrogenase subunit 2 |
| NADH dehydrogenase subunit 3 |
| NADH dehydrogenase subunit 4 (EC:1.6.99.3) |
| NADH ubiquinone oxidoreductase chain A (EC 1.6.5.3) |
| NADH-ubiquinone oxidoreductase chain B (EC 1.6.5.3) |
| NADH-ubiquinone oxidoreductase chain C (EC 1.6.5.3) |
| NADH-ubiquinone oxidoreductase chain D (EC 1.6.5.3) |
| NADH-ubiquinone oxidoreductase chain E (EC 1.6.5.3) |
| NADH-ubiquinone oxidoreductase chain F (EC 1.6.5.3) |
| NADH-ubiquinone oxidoreductase chain G (EC 1.6.5.3) |
| NADH-ubiquinone oxidoreductase chain H (EC 1.6.5.3) |
| NADH-ubiquinone oxidoreductase chain I (EC 1.6.5.3) |
| NADH-ubiquinone oxidoreductase chain J (EC 1.6.5.3) |
| NADH-ubiquinone oxidoreductase chain K (EC 1.6.5.3) |
| NADH-ubiquinone oxidoreductase chain L (EC 1.6.5.3) |
| NADH-ubiquinone oxidoreductase chain M (EC 1.6.5.3) |
| NADH-ubiquinone oxidoreductase chain N (EC 1.6.5.3) |
| Proline dehydrogenase (EC 1.5.99.8) (Proline oxidase) |
| Putative formate dehydrogenase oxidoreductase protein |
| Putative NADP-dependent oxidoreductase PA1648 |
| Putative Zn-dependent oxidoreductase PA5234 |
| Quinone oxidoreductase (EC 1.6.5.5) |
| soluble fumarate reductase, cytoplasmic |
| Succinate dehydrogenase cytochrome b-556 subunit |
| Succinate dehydrogenase cytochrome b560 subunit, mitochondrial precursor |
| Succinate dehydrogenase flavoprotein subunit (EC 1.3.99.1) |
| Succinate dehydrogenase iron-sulfur protein (EC 1.3.99.1) |
| ubiquinol cytochrome C oxidoreductase, cytochrome C1 subunit |
| Ubiquinol--cytochrome c reductase, cytochrome B subunit (EC 1.10.2.2) |
| Ubiquinol-cytochrome C reductase complex core protein I, mitochondrial precursor (EC 1.10.2.2) |
| Ubiquinol-cytochrome C reductase complex ubiquinone-binding protein QP-C (EC 1.10.2.2) |
| Ubiquinol-cytochrome C reductase iron-sulfur subunit (EC 1.10.2.2) |
| V-type ATP synthase subunit A (EC 3.6.3.14) |
| V-type ATP synthase subunit B (EC 3.6.3.14) |
| V-type ATP synthase subunit D (EC 3.6.3.14) |
