# Supplementary figures and images for: Microbial enrichment and gene functional categories revealed on the walls of a spent fuel pool of a nuclear power plant
Source: PLoS One. 2018 Oct 4;13(10):e0205228. doi: 10.1371/journal.pone.0205228 (PMC6171911; doi:10.1371/journal.pone.0205228)

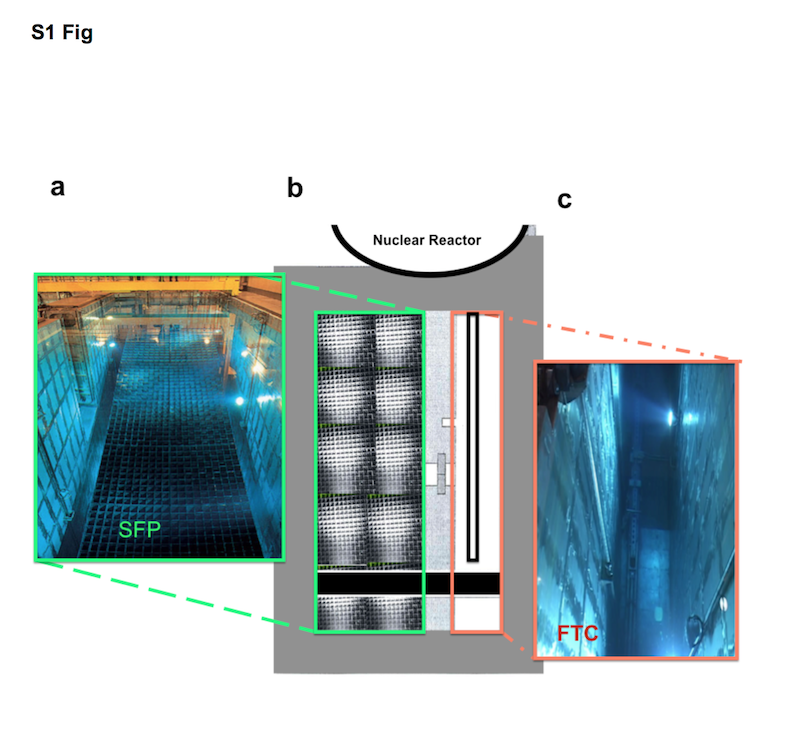

Supplement: S1 Fig — (a) SFP photograph, showing the spent nuclear fuel at the bottom of the pool; (b) scheme of the compound that stores the spent nuclear fuel, left the SFP; and to the right the FTC; (C) FTC photograph showing the robot arm, at the bottom, used to transfer nuclear fuel to the reactor or from the reactor to the SFP. SFP and FTC facilities are massive concrete structures with a corrosive resistant stainless-steel ASTM A240, type 304L liner material to guarantee structural quality. The nuclear power plant Angra 1 in Rio de Janeiro, Brazil has a pressurized water reactor (PWR) that uses 121 fuel elements (256 rods, 4 meters high each) of enriched Uranium-235. After 12 months, part of the fuel in the core of the reactor is replaced. The spent fuel remains thermal active and radioactive and is transferred through a fuel transfer tube to 1252 storage cells of super compact racks at the bottom of a spent fuel pool (SFP). The quality of the water must meet strict requirements of purity and clarity allowing operators to handle irradiated fuel elements. (TIFF) [file pone.0205228.s005.tiff]

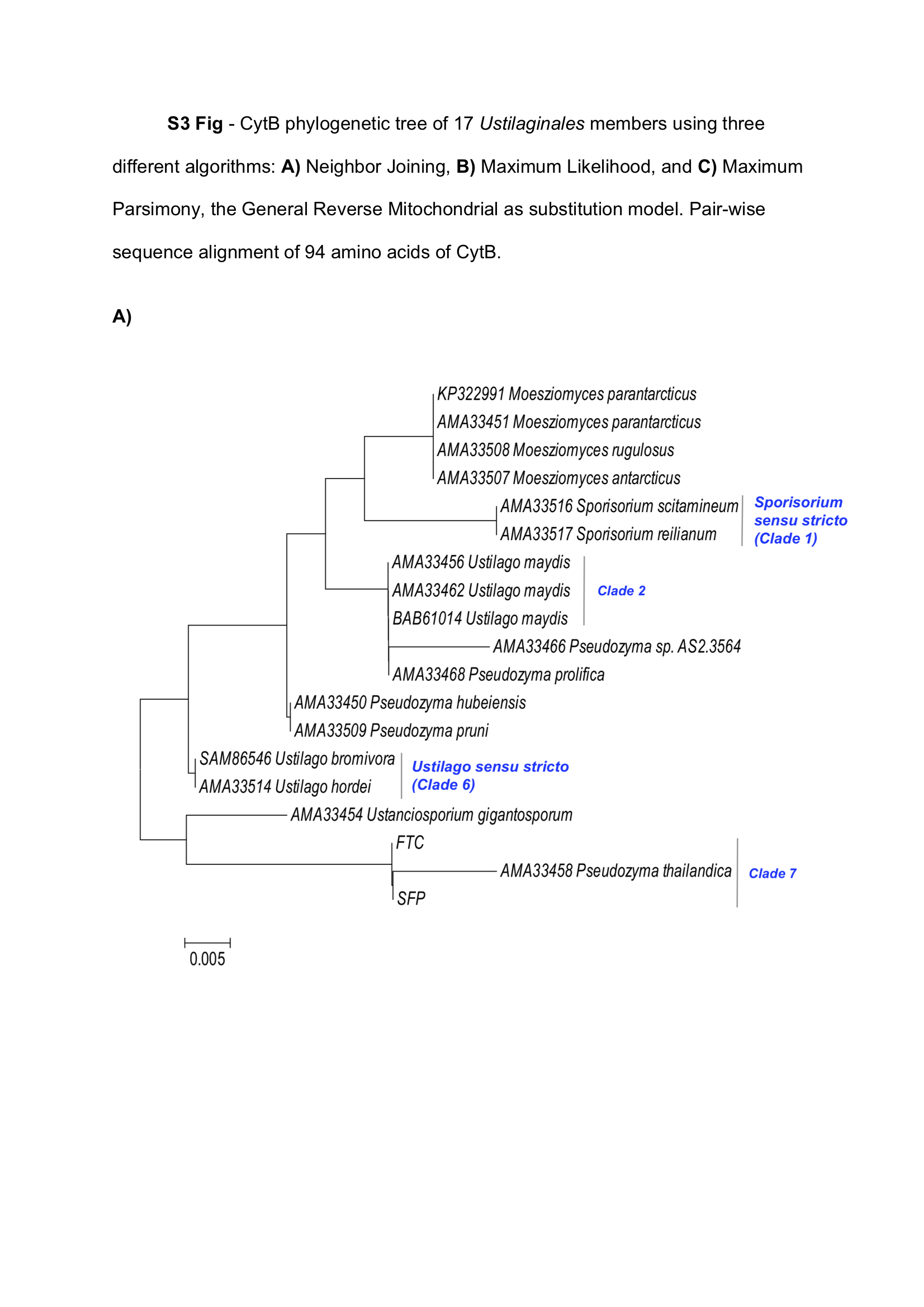

Supplement: S3 Fig — cytb phylogenetic tree of 17 Ustilaginales members using three different algorithms: A) Neighbor Joining, B) Maximum Likelihood, and C) Maximum Parsimony, the General Reverse Mitochondrial as substitution model. Pair-wise sequence alignment of 94 amino acids of cytb. (TIFF) [file pone.0205228.s007.tiff]

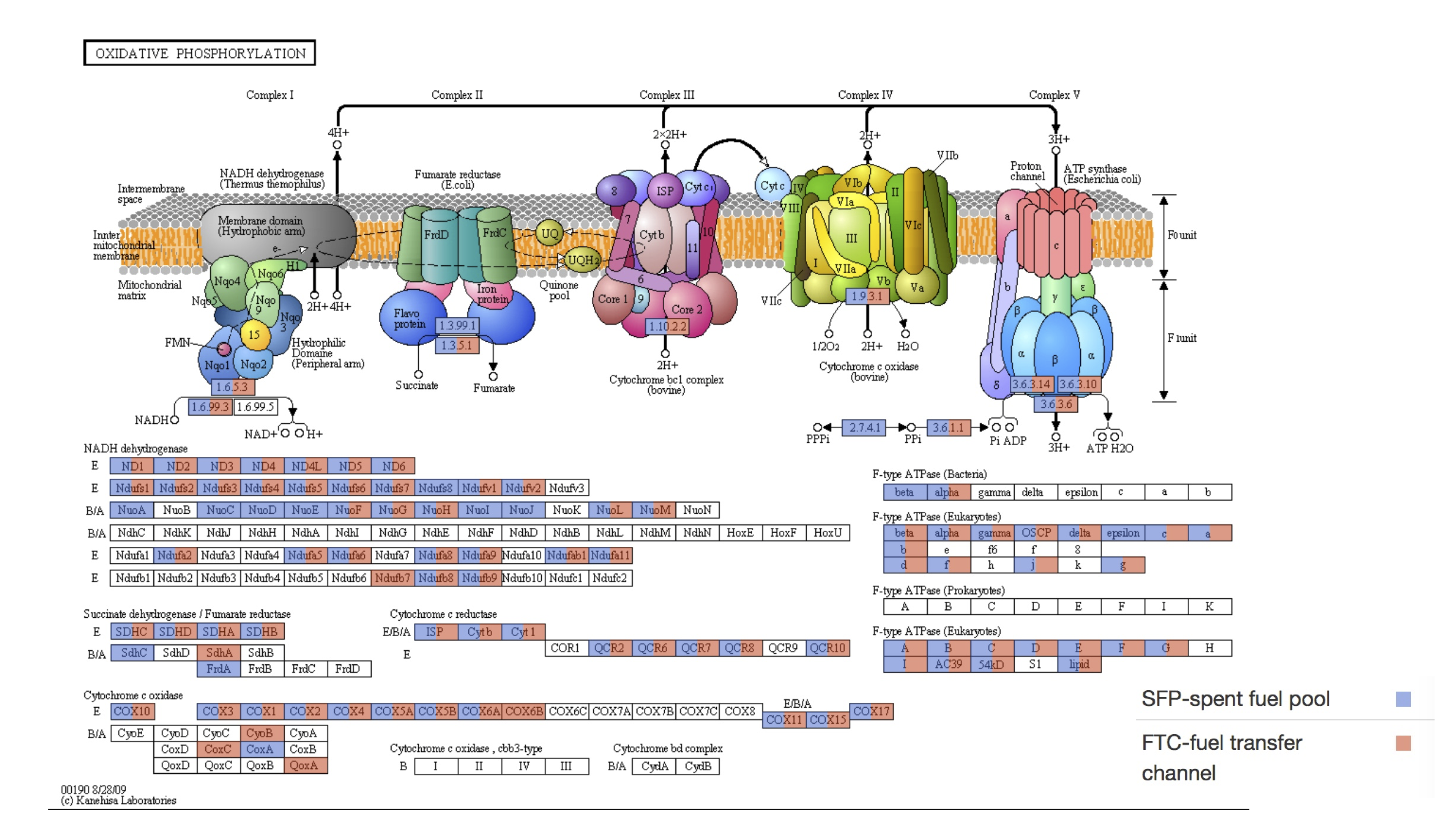

Supplement: S4 Fig — (TIFF) [file pone.0205228.s008.tiff]
